# Why Functional Pre-Erythrocytic and Bloodstage Malaria Vaccines Fail: A Meta-Analysis of Fully Protective Immunizations and Novel Immunological Model

**DOI:** 10.1371/journal.pone.0010685

**Published:** 2010-05-19

**Authors:** D. Lys Guilbride, Pawel Gawlinski, Patrick D. L. Guilbride

**Affiliations:** 1 Deutsches Krebsforschungszentrum (DKFZ), Heidelberg, Germany; 2 Member of the Royal College of Veterinary Surgeons (MRCVS), London, United Kingdom; Federal University of São Paulo, Brazil

## Abstract

**Background:**

Clinically protective malaria vaccines consistently fail to protect adults and children in endemic settings, and at best only partially protect infants.

**Methodology/Principal Findings:**

We identify and evaluate 1916 immunization studies between 1965-February 2010, and exclude partially or nonprotective results to find 177 completely protective immunization experiments. Detailed reexamination reveals an unexpectedly mundane basis for selective vaccine failure: live malaria parasites in the skin inhibit vaccine function. We next show published molecular and cellular data support a testable, novel model where parasite-host interactions in the skin induce malaria-specific regulatory T cells, and subvert early antigen-specific immunity to parasite-specific immunotolerance. This ensures infection and tolerance to reinfection. Exposure to *Plasmodium-*infected mosquito bites therefore systematically triggers immunosuppression of endemic vaccine-elicited responses. The extensive vaccine trial data solidly substantiate this model experimentally.

**Conclusions/Significance:**

We conclude skinstage-initiated immunosuppression, unassociated with bloodstage parasites, systematically blocks vaccine function in the field. Our model exposes novel molecular and procedural strategies to significantly and quickly increase protective efficacy in both pipeline and currently ineffective malaria vaccines, and forces fundamental reassessment of central precepts determining vaccine development. This has major implications for accelerated local eliminations of malaria, and significantly increases potential for eradication.

## Introduction

### The malaria vaccine paradoxes

A solitary subunit vaccine marginally [1], [2], [3], [4], [5], [6] protects children in endemic areas [7], [8] against malaria, and only partially protects infants [9], [10], [11], similarly to malaria-naïve adults [12], [13]. Adults in endemic areas remain unprotected [14], [15]. These data crystallize the paradoxes central to 80 years of malaria vaccine research. Endemic populations display T cell [16], [17], [18], [19], [20], [21], [22], [23] and antibody [24], [25], [26], [27], [28], [29] responses to all malaria lifecycle stages and rapidly acquire immunity to bloodstage parasites, mitigating adult disease and death [29], [30], [31], [32], [33]. Immunity to earlier skinstage parasites however does not develop, and endemic populations remain tolerant to continual reinfection [32], [33], [34] remaining at risk for severe malaria should immunocompetence weaken. Similarly, potentially protective [17], [35], [36], [37], [38] T cell responses elicited by diverse attenuated-parasite [20], [39], [40], [41], [42], [43], [44], [45], [46], [47], [48], [49], [50], [51] and subunit [52], [53], [54], [55], [56], [57], [58], [59], [60], [61], [62], [63], [64], [65], [66], [67], [68], [69] malaria vaccines, and laboratory infections [35], [42], [51], [70], [71], [72], [73], [74], [75], [76], [77] provide sterile immunity [38], [47], [78], [79], [80] to infection, yet are ineffective in endemic populations [14], [15], [81], [82], [83], [84] and are effectively blocked by the parasite [85]. Ostensibly, protection is blocked only in endemic areas, implying a conditional difference between laboratory and field infections which systematically triggers an immunological block to vaccine function in the field. This rationale pinpoints an activatable immune mechanism which blocks existing T cell responses. Obvious candidates are the normal immune mechanisms suppressing autoimmunity and allergy, or self- and nonself-tolerance. These mechanisms centre largely around suppressive function of regulatory T cell subsets (Tregs) [86], [87]. Activated natural (nTreg) and induced (iTreg) Tregs suppress effector T cell [86], [87] and B cell responses [88], [89], [90], [91], [92] and tolerize dendritic cells (DC) [93] to maintain self-tolerance and regulate inflammatory responses to injury [94], tissue grafts [95], [96], [97], pathogens and allergens [98], [99], [100].

A major regulatory immune organ, rich in regulatory T cells [101], [102], is human skin. This suggests the initial path of malarial infection will profoundly affect systemic host responses to subsequent lifecycle stages. Most experimental infections bypass the skin entirely. Natural malaria infection however, starts in the skin [103], [104], [105], [106], [107]. Infected mosquitoes inject motile [108] skinstage (sporozoite) parasites; within minutes, a few migrate to proximal lymphatic vessels and skin-draining lymph nodes (LN) [107]. Another few [107] invade blood vessels, rapidly [109], [110], [111] migrating to the liver; the remainder linger in the skin [107]. Liver invading parasites differentiate [112] and multiply (for days) asymptomatically [32] until bloodstage-filled vesicles bleb [113], [114] into the blood, causing the systemic inflammatory reaction [115], immunologically similar to sepsis [115], underlying initial symptoms of clinical malaria. We compile the immunobiology of Anopheline mosquito bites and human skin, with the molecular and behavioural characteristics of skinstage parasites, to show that natural infection leads inevitably to systemic immunotolerance. We further show, via comprehensive meta-analysis of fully protective vaccine trials, that bypassing or disrupting natural parasite-host immune interactions in the skin profoundly affects host responses to vaccine antigens, and leads to protective immunization against malaria.

## Methods

### Literature Searches for completely protective vaccine trials, for meta-analysis

Searches were performed following MOOSE (Meta-analysis of Observational Studies in Epidemiology) guidelines for conduct and PRISMA (Preferred Reporting Items for Systematic Reviews and Meta-analysis) reporting protocol for Systematic Reviews and Meta-analysis [116]. Studies showing complete protection to malaria challenge in mammals were identified by searching the PubMed database (www.ncbi.nlm.nih.gov/pubmed/) with search terms: malaria OR plasmodium & (complete &) protect* OR immuni* OR vaccine OR human/man/chimpanzee/monkey/mouse/rat/rabbit/dog/goat/OR

Aidoo/Ballou/Beaudoin/Clyde/Corradin/Daubersies/Druilhe/Doolan/Egan/Good/Herrington/Hafalla/Hill/Hoffman/Hollingdale/Heussler/Kappe/Kester/Krzych/Khusmith/Langhorne/MacColm/McCarthy/Marsh/Mazier/Miller/Most/

Matuschewski/Nardin/Nussenzweig/Playfair/Plebanski/Orjih/Orton/Patarroyo/

Renia/Rieckmann/Riley/Rodrigues/Sauerwein/Sedegah/Schofield/Siddiqui/

**Figure 1:**
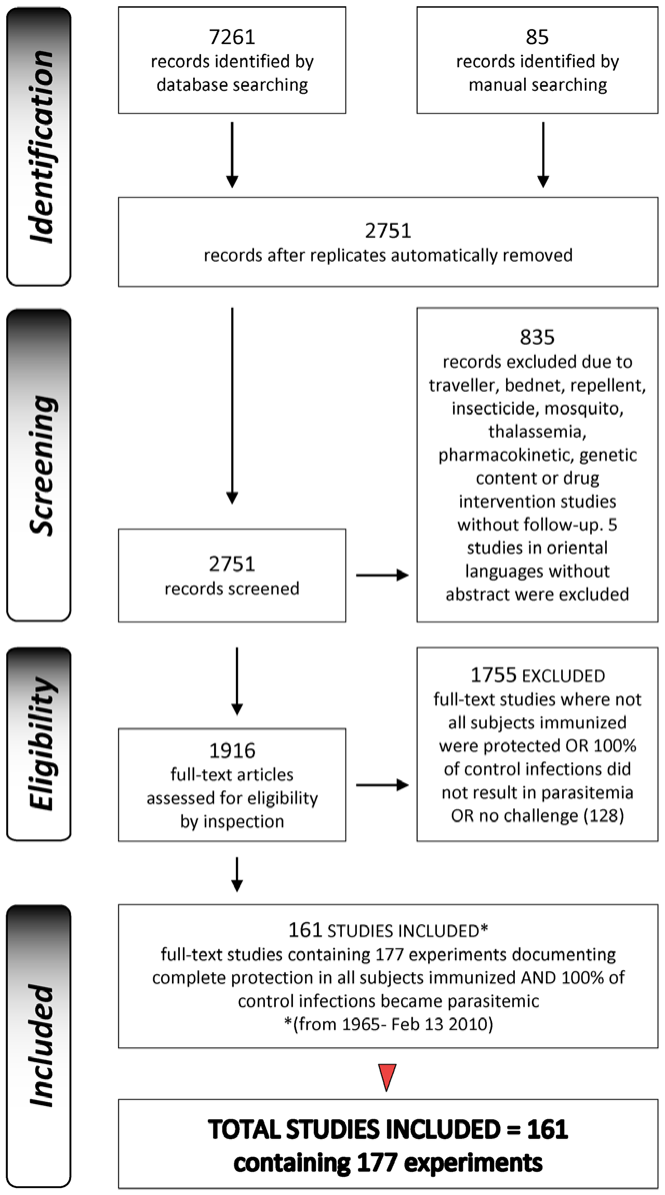
PRISMA Flow Diagram showing inclusion/exclusion criteria for studies documenting complete protection 1965-February 2010.

Snounou/Tartz/Tsuji/Urban/Vanderberg/Vaughan/Weiss/Weidanz/White/Yoshida/Zavala/&/OR year (1965 – 2009). Searches were performed by adding two or three qualifiers at a time to the basic search string: [“malaria” OR “plasmodium” AND protect*” OR “immuni*”], and rerunning the search each time. We ran searches without, then with, qualifier term “complete” to allow wide retrieval sensitivity. Retrieved records were combined and replicates removed. Compiled single records were then screened by search term and abstract perusal for traveller, bednet, thalassemia, genetic, pharmacokinetic, drug intervention, insecticide, repellent or mosquito physiology content without reinfection follow-up, and excluded; we also contacted authors to verify conditions where necessary. This search strategy, (outlined in Figure 1), provided meticulous coverage, as determined by random spot-checks for coverage. Last complete database searches were carried out 9–13 September 2009; final update searches were run 13 February 2010. We also examined cited reference lists in studies and reviews identified. Late-breaking studies were manually added to this final database.

### Selection of studies for meta-analysis data–Inclusion/exclusion criteria

Inclusion criterion: Identified full-text immunization studies were then checked individually by full-text inspection for experimental immunization and challenge conditions, and results of challenge, and sorted with regard to complete protection criteria (defined below). Immunization studies showing complete protection of all immunized subjects and infectivity in all control infections (in any part of the study), were included in a final dataset for analysis (Defined in Figure 1; listed in Table S1). No other inclusion criterion was used. Studies in English, French, Spanish, Portuguese and German were assessed, and sufficient discriminatory data was available in English abstracts, for most studies identified published in further languages. Studies in Oriental languages without abstract (5) were not evaluated. Included studies were further sorted for analysis according to experimental data as described below in Validity and Sorting Criteria. Exclusion criteria: Studies showing incomplete protection in immunized subjects, or incomplete control infectivity, were excluded. No other exclusion criterion was used on the immunization studies identified by the search method described.

### Validity Assessment and Sorting Criteria

**Figure 2:**
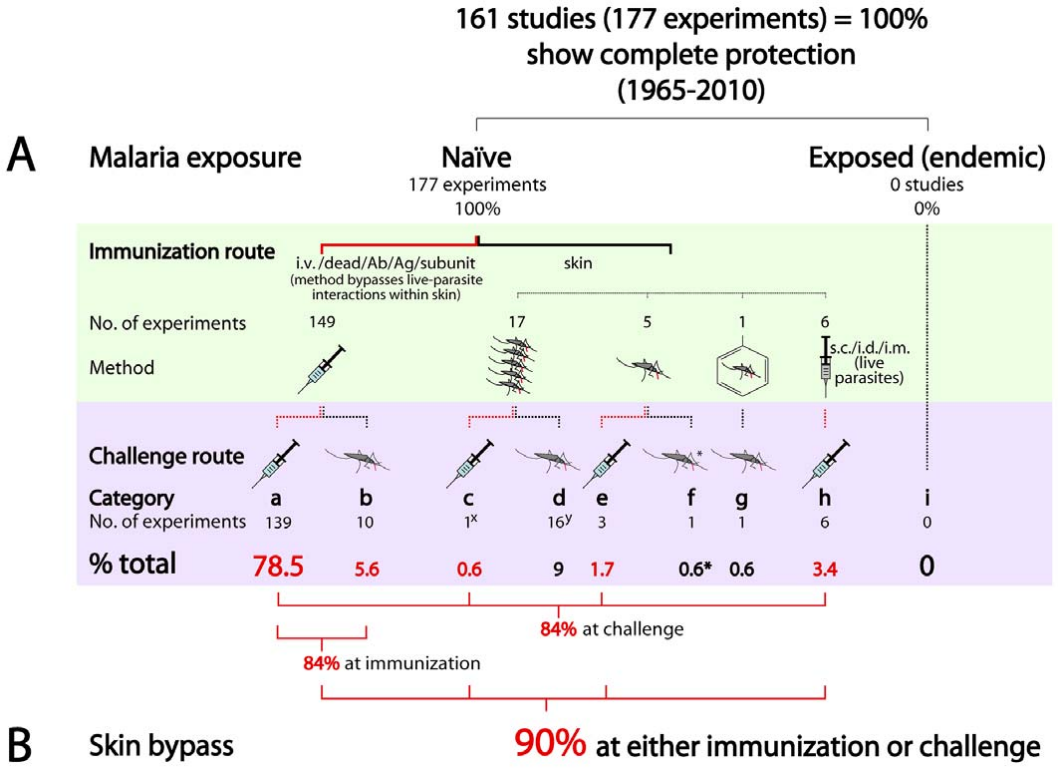
Protective vaccination physically bypasses skin at immunization or challenge (90%) or involves skin immunomodulation (10%). A. Exposure to parasites in the skin coincides closely with vaccine failure. Green background- immunization procedures. Lilac background- challenge procedures and percent of total experiments showing complete protection (% total) formed by a subset of studies (category) using a given experimental procedure (categories a-i; supporting data in references listed below). Inclined syringe- administration route is intravenous (i.v.) for live parasites, or, the method does not involve live parasites, but uses dead parasites or purified antigen, antibody, or recombinant DNA (dead/Ag/Ab/subunit) and therefore bypasses parasite interactions with host skin. Multiple mosquitoes- live parasites administered by multiple simultaneous mosquito bites. Single mosquito- live parasites naturally transmitted by 4–15 bites. Mosquito in aromatic ring- live parasites administered by 12–15 bites/session with chloroquine. Vertical syringe- live parasites delivered subcutaneously (s.c) or intradermally (i.d.) or intramuscularly (i.m.); (i), uncontrolled exposure to endemic mosquitos. B. Protective immunization physically bypasses the skin at either immunization or challenge in 90% of cases (a,b,c,e,h). Protective immunization which transits skin during immunization (c,d,e,f,g,h) either: bypasses the skin physically at challenge (c,e,h); or, involves skin immunomodulation during immunization (d,g, 10% of cases). Within A and B: Red lines and numbers- experiment bypasses parasite-skin interactions at stage indicated by red lines; black lines and numbers- parasites interact with skin at stage indicated by black lines. Asterisk (*)- immunization via unmodified skin, limited to less-virulent *P. berghei* (f). Skin bypass- method physically avoids live parasite interactions in the host skin. Malaria exposure- skin exposure to infected mosquito bite before first immunization; naïve- no pre-exposure; exposed (endemic)- chronic exposure. x- this study shows complete protection of 40 of 41 mice challenged. y: one person in one study [117] was infected one time via the skin prior to protective immunization and was therefore moderately tolerized. Data pertaining to experimental categories (a–h): a:[35], [42], [47], [49], [50], [51], [57], [68], [70], [71], [72], [73], [74], [75], [76], [78], [85], [124], [125], [126], [128], [129], [130], [133], [137], [138], [139], [150], [151], [166], [169], [171], [173], [185], [267], [335], [336], [337], [346] [355], [356], [357], [358], [359], [360], [361], [362], [363], [364], [365], [366], [367], [368], [369], [370], [371], [372], [373], [374], [375], [376], [377], [378], [379], [380], [381], [382], [383], [384], [385], [386] [387], [388], [389], [390], [391], [392], [393], [394], [395], [396], [397], [398], [399], [400], [401], [402], [403], [404], [405], [406], [407], [408], [409] [410], [411], [412], [413], [414], [415], [416], [417], [418], [419], [420], [421], [422], [423], [424], [425], [426], [427], [428], [429], [430], [431], [432], [433], [434], [435], [436], [437], [438], [439], [440], [441], [442], [443], [444], [445], [446], [447], [448], [449], [450], [451]. b: [47], [48], [124], [128], [129], [138], [171], [387], [452], [453]. c: [124]. d: [36], [44], [118], [119], [120], [121], [122], [123], [124], [134], [135], [136], [172], [454], [455], [456]. e: [111], [132], [457]. f: [127]. g: [163]. h: [48], [111], [388], [458], [459]. Studies containing data for multiple relevant experimental conditions are referenced accordingly in each appropriate category. Multiple experiments contributed by a single study are indicated beneath study reference number (eg. reference 124 X2) in Supplementary Table S1. (Meta-analysis data extended reference list).

Complete protection criteria: bloodstage parasites undetectable after challenge in all immunized subjects AND 100% of non-immunized controls became parasitemic. Experiments documenting complete protection were further sorted into 8 categories, (a–h), defined first by route and method of immunization, and then route of challenge. Experimental data publications are listed by category in Supplementary Table S1. The route and conditions defining immunization in each category are represented graphically in Figure 2 (and in written form in Supplementary Table S2) on green background, and explained in the legends to Figure 2 and Supplementary Table S2. The route of challenge, either intravenous or via moquito bite, is indicated graphically on lilac background, (in written form in Table S2) and explained in legends for Figure 2 and Table S2.

### Data Abstraction

Final database searches as described were performed separately twice during a three month period (June-September 2009), and once in February 2010, by the same person, and a further search performed once by a second person. Four separate results databases were combined with longterm manual search archives from over 4 years, and replicates removed. Assessment of compliance with complete protection criteria for the final dataset was reconfirmed in all cases by one person. From this final dataset, by full-text inspection, we retrieved specific experimental data regarding method and route of immunization, and method and route of challenge.

### Analysis/Summary measures

We measured the percentages of our dataset of fully protective vaccination experiments, that involved either absence, or presence, of live parasite interaction within the skin, during immunization, during challenge, or during both.

## Results

### 1. Selection of studies for vaccine trial meta-analysis

Literature search, final selection strategy and data retrieval were designed to return data completely unlinked to and irrespective of any criterion beyond complete protection. Searches targeted any immunization, in any mammal, generating a comprehensive database of immunizations from 1965-February 2010. Random spot-checks confirmed meticulous coverage. Search method, inclusion/exclusion, assessment and sorting criteria are fully described in the Methods section. The probability of missing any important immunization study is therefore finite but exceedingly small. The probability of missing an entire class of vaccines is nil. Literature searches and resulting datasets of factors contributing to protective vaccine function were therefore comprehensive, and completely unfocused and unbiased with regard to parasite life-cycle stage, antigen character, proposed immune mechanism, mode of application or any other criterion beyond complete protection. Data for analysis (specific experimental data regarding method and route of immunization, and method and route of challenge) was included according to a rigorous definition of complete protection.

We identify 1916 immunization studies performed worldwide between 1965 and February 2010 (Fig. 1), and retrieve an unambiguous, comprehensive and unbiased data set of 177 experiments in 161 publications documenting completely protective immunization, versus 1627 nonprotective or partially protective immunization results (Fig. 1).

### 2. Vaccine trial data implicates the skin in vaccine failure

#### 2-i. Meta-analysis shows complete protection against natural challenge, by immunization via unmodified skin, does not occur

Completely protective vaccinations (177 experiments, Fig. 2; see also Supplementary Table S2, and Fig. 1) employing diverse vaccines, fall into 8 categories (Fig. 2a–h) according to method, and routes of immunization and challenge. These reveal that, unlike endemic populations (Fig. 2i), all protected subjects (Fig. 2a–h, with verifiable exception of one individual [117]) are malaria-naïve (unexposed to malaria-infected mosquitoes) prior to first immunization (Fig. 2A). Most protective vaccinations also physically bypass live parasite interactions in the skin at either immunization or challenge (90%, Fig. 2B, a,b,c,e,h). Bypass is either by intravenous (i.v.) injection, or use of dead parasites, purified antibody or protein, or recombinant DNA, at immunization (84%, Fig. 2a,b), or i.v. injection at challenge (84%, Fig. 2a,c,e,h), usually by i.v. injection at both (78.5% Fig. 2a). Skin-based immunization (via infected mosquito bite), completely protective against virulent natural challenge (Fig. 2d,g) in humans [118], [119], [120], [121], [122], [123] and mice [124] occurs only under conditions significantly altering the immune context in the skin during immunization. These immunomodulary conditions are illuminating and are examined in detail in Sections 3 and 4, and entirely account for the remaining 10% of completely protective immunization.

#### 2-ii. Partially and non-protective studies strongly suggest parasite skin interactions actively depress host immunity

Otherwise, in mice, live-parasite immunization “via the skin” (this includes subcutaneous injection (s.c.) which bypasses, but inevitably contaminates, the skin) is usually non-protective [125], [126]. Where documented, skin-based immunization provides complete [127] (Fig. 2f) or substantial [124], [128], [129] immunity only against less infective *Plasmodium berghei* natural challenge, but not against ∼100-fold more infective [130] *P. yoelii* natural challenge [127], [128]. Intravenous immunization with *P. yoelii* protects completely against intravenous challenge [128], [129], but mosquito delivered [128], [129], or high-dose injected intradermal [128] immunization does not. This suggests skin-immunization generates immunity less efficiently [124], [128], or increases intrinsic parasite infectivity [124], [128], [131].

However, after identical intravenous infective challenge (bypassing the skin) [128], diminished immunity with high dose skin immunization (compared to intravenous) [128] must derive from deficient host responses (activated less efficiently, or actively depressed, or both), not parasite-intrinsic changes.

Therefore, since skin immunization with less infective [130] *P. berghei* protects completely against intravenous infection (20,000–50,000 parasites) [124], [132], but not against a 20–200X lesser skin challenge (10 bites) [124], (roughly 250–1000 parasites [107], [110]) the data argue strongly for parasite-skin interactions increasing host susceptibility by actively depressing host immunity.

#### 2-iii. Immunity generated via unmodified skin is easily broken

Intravenous mouse [128] and primate [68], [133] attenuated-sporozoite immunizations withstand repeated intravenous challenge. Immunity generated by live parasites via skin however, is reversed by small increments (5 additional bites, or 125–500 more parasites) in natural challenge dose [124], [128], but withstands heavy intravenous challenge (20,000–100,000 parasites) [124], [125], [126], [128], [132] (see also Fig. 2c,e,h). Likewise, skin-generated immunity in humans [119], [134] (see also Fig. 2d) despite immunizing doses 100-fold greater than challenge, succumbs to increased [119], [134], and usually, sequential [119], [123], [134], [135], [136] natural challenge. Immunity generated transiting skin, therefore, is marginal, and reversible.

#### 2-iv. Bloodstage vaccines do not protect against challenge via the skin

Finally, intravenous immunization with bloodstage parasites [76], [77], [137], [138], fully protects against intravenous bloodstage challenge in humans [77] and monkeys [137] and both bloodstage [138], [139] and sporozoite [76] intravenous, but not mosquito-bite, challenge [138] in skinstage-naïve mice. Similarly, major bloodstage-antigen vaccines (eg. MSP-1_42_, AMA-1), show strong antibody-correlated [61], [67], [140] efficacy against symptomatic malaria after intravenous challenge in monkeys [67], [140] and induce similar antibody responses in people from endemic areas [83], [141], [142]. Protective efficacy against infection however, is negligible, despite some evidence of reducing risk of symptom severity and parastaemia density [143], [144]. No bloodstage antigen in over 16 trials and 10,300 humans vaccinated to date, protects against infection by mosquito bites [83], [84], [142], [143], [145], [146], [147] (and Figure 2i).

#### 2-v. Summary: Vaccine trial data implicates the skin in vaccine failure

Collectively, these data show that in malaria-naïve subjects, (which excludes bloodstage immunosuppressive effects) live-parasite immunization transiting unmodified skin is inefficient. Immunity diminishes after unmodified skin-parasite interactions and is significantly less robust generated via skin than if intravenously. The bulk of experimentation shows immunization avoiding parasite-skin interaction withstands heavy, repeated intravenous challenge, but only limited challenge transiting the epidermis. Importantly, the data imply parasite-skin interactions actively diminish host protective responses. Avoiding parasite/host skin interactions during both immunization and challenge however, associates solidly with immunity.

### 3. Altered immune context in the skin during immunization protects against natural challenge and suggests a skin-linked immunosuppressive mechanism

#### 3-i. Immunization under pro-inflammatory skin conditions confers protection

Complete human protection with irradiation-attenuated [40], [118], [120], [122], [136] *P. falciparum* or *P. vivax* requires 1000 or more mosquito bites [120], [134] (usually 80–240 bites/session). This causes coalescing skin inflammation [40], [134], lasting several hours [134]. Immunity is reversed by relatively small increases [119], [134] in challenge dose, as for mice [124], [128]. Fewer total immunizing bites (<1000) are not reliably [134], or (<700) not at all [52], [135] protective when delivered in low density bites/session [52], [118], [119], [135], or with strongly anti-inflammatory topical cream [40] and/or heavier parasite irradiation [40], [52]. However, 440 infected bites, delivered with additional uninfected bites, increasing bite density, is protective [123]. Rather than parasite dose alone [134], [148], therefore, protection appears influenced by degree of parasite attenuation (limiting liverstages [149]), density of simultaneous bites, and pro-inflammatory local context.

#### 3-ii. Immunization via unmodified skin suppresses available protective responses

Systemic proinflammatory context confers resistance to malaria infection in mice [150], [151], [152] and correlates strongly with human resistance [153], [154], [155]. In uninflamed mouse skin, increasing immunizing dose from 2 mosquito bites (roughly 50–200 parasites [107], [110]) to 4 bites, significantly increases parasite-specific (CD8+) T cell responses [79]. More immunizing bites yield no further increases [79]. Yet, 100-fold higher immunizing doses (20 000 parasites) delivered directly (i.v.) to the liver, provide almost twice the protection of 10 000 parasites [47], proportionally increasing specific T cell responses [79]. This reveals higher protective responses to direct liverstage infection are available, which become unavailable when parasites transit uninflamed skin. An intravenous immunizing dose completely protective against i.v. challenge of 20,000 parasites, but not against 10 bites [124] (20-80-fold fewer parasites), is further consistent with skin-linked immunosuppression.

These data suggest inflammatory skin context potentiates immunization via skin, implying inflammation relieves a skin-linked suppression of immunity to liverstage malaria unassociated with prior bloodstage infection.

#### 3-iii Skin-immunization under chloroquine (CQ) immunomodulation also confers protection

CQ, a widely immunomodulatory 4-aminoquinolone, accumulates preferentially in human skin and lymphocytes [156], to levels 200–20,000 -fold those in the liver, and remains in human skin, (not plasma) for 6 months [157]. Prophylactic *in vivo* concentrations (100–500 ng/ml liver plasma [157], [158]) inhibit antigen presentation in human antigen-presenting dendritic (DC) and B cells *in vitro* [159], [160], [161].

Immunization via skin with a drastically lower (but normally 100% infective [162]) dose of unattenuated *P. falciparum* (15 infected bites/session, x3), if co-administered with prophylactic chloroquine (CQ), instead protects malaria-naïve individuals from challenge (5 infective bites) [163]. CQ provides greater immunity than parasite attenuation: considerably greater immunizing doses of irradiated parasites without CQ do not protect mice [47] or humans [134] against natural challenge. CQ treatment either allows greater and/or antigenically broader immune responses consequent to robust expression of liverstage antigens [47], [163], [164], or lowers a barrier to immunity, or both. We examine the evidence for both, below.

### 4. Protective effects of skin inflammation and CQ immunomodulatory mechanisms implicate early, skin-induced Tregs in systemic immunotolerance

#### 4-i. The liverstage antigen concept for protective immunity

That protection requires robust expression of liverstage-antigens has significant correlative support. Primaquine (PQ) eliminates all forms of liverstage parasites [47], [165] and concomitantly abrogates protection [165], [166]; incremental parasite irradiation increasingly limits parasite liverstage proliferation [149], [165] and corresponds to decreasing antigen synthesis [167] (implying decreased antigen presentation) and diminished protectivity [165], [167], [168], [169], [170]. CQ attenuation however, does not affect parasite liver stages [47], allowing fullscale liver infections, abrogated after emerging from the liver [47]. These data imply robustly expressed mid-lateliverstage parasite antigens provide immunity [47], [170] to subsequent natural challenge.

#### 4-ii. The liverstage antigen concept does not explain all the data

Although widely accepted [163], this concept does not reconcile important data. Genetically attenuated liverstage parasites (p52/36, delivered i.v.), persisting in hepatocytes less than 6 hrs [171] (minimizing antigen presentation) nonetheless protect against stringent *P. yoelii* [171] natural challenge. Similarly, protective responses to liverstage infection (delivered i.v.) are swift [46], [111], but do not increase with prolonged antigen presentation [79]. Stable, protective [172] memory T cell populations to skinstage parasites are induced and maintained by exposure to sporozoite and bloodstage parasites in skinstage immunized, (malaria-naïve) people [172]. Finally, endemic populations remain susceptible to reinfection, despite multiple fullscale liverstage infections, widespread use of CQ in adults, and evidence for unimpaired development and recall of liverstage-cognate memory responses during [80], [173], [174] and after [174] bloodstage infection. These discrepancies suggest CQ co-administered with skin-infecting parasites in malaria-naïve people, confers protection in some way beyond allowing a fullscale liverstage antigenic repertoire.

#### 4-iii. CQ inhibits antigen presentation pathways vital to Treg induction in skin

CQ disrupts all MHC II antigen presentation [157], [175], [176] to CD4+ T cells. CQ also specifically blocks [160] the rapid, seconds-to-15-minute [159] noncanonical MHC I recycling pathway [160], [161] for extracellular antigen cross-presentation to cytotoxic (CD8+) T cells, which in human plasmacytoid-DC (pDC) [160] enables very rapid cytotoxic responses [160]. CQ does *not* block classical MHC I antigen cross-presentation [161], which takes 6-18 hrs [160] for optimal presentation.

Therefore, only slow, classical MHC I cross-presentation, (efficiently functional in human skin-resident epidermal Langerhans DC (L-DC) [177], [178], dermal DC (d-DC) [179] and other immune and parenchymal cells [180] such as traversed [181] skin or malaria-infected liver cells [111], [182]), is available to generate protective cytotoxic (CD8^+^, MHC I) responses to malaria antigens presented at the skinstage [18], [28], [183], [184] in humans. In agreement, protection provided by attenuated sporozoites against intravenous challenge in mice, requires MHC I cross-presentation [185]. Also, in humans, one therapeutic dose of CQ strongly inhibits CD4+ T cell responses while strongly inducing CD8+ effector responses *in vivo* to a particulate viral antigen [186]. Malaria parasites constitute particulate antigen. Evidently, CQ treatment will affect antigen presentation in the skin during the earliest stages of malaria infection: the mosquito bite. Antigen-specific responses dependent on rapid cross-presentation of exogenous MHC I, and all MHC II antigen presentation, including any regulatory T cell induction or activation, will be blocked. CQ therefore, affects the balance of earliest host immune responses triggered to malaria antigens, which are first encountered in the skin.

#### 4-iv. Normal host response to mosquito-bite inflammation triggers tolerizing cascades, facilitating parasite-cognate Treg induction early in infection

Mosquito bites cause severe local or systemic allergic inflammation [187], unless rapidly dampened by *in situ* suppressive activity induced in antigen-cognate T cells (iTregs) [91]. Human skin is rich (∼100,000 per cm^2^) [101], [102] in suppressive [102], skin-resident [101], [102], circulating [188], [189] and LN-homing [102] nTregs, and local inflammation, which recruits DCs and T cells [91], [190] preferentially increases Treg infiltration [91], [191], [192]. Tregs further accumulate at inflammatory sites [91], [193], [194] via DC (antigen-presenting) dependent and independent [102] mechanisms.

In skin, sunlight and allergen induced inflammations cause keratinocyte signalling molecules [195] to interact with densely interwoven epidermal [196] L-DC. This increases antigen scavenging [195] in L-DC, and induces L-DC to generate *de novo,* and proliferate, antigen-specific Tregs [195]. These skin-triggered Tregs repress allergic inflammatory reactions both locally, in skin and draining LNs, and systemically [195].

Mosquito saliva allergens [187], in sensitized subjects [152] such as endemic populations also trigger [197] almost instantaneous [198] inflammatory extravasation [152], [187], [190], IgE-mediated [199] and independent mast-cell TNF-α [190] and local IFN-γ and inflammatory cytokine secretion [152], [187], [190]. This induces chemokines [200] enabling [201] local skin inflammations to rapidly recruit leukocytes [152], [187], [190], including circulating immature monocyte-derived DC (mDC) [200], pDC [200], [202], [203], [204], [205], [206], [207] not normally present in skin, and skin-resident dDC and immature L-DC [200]. While immature L-DC are initially tolerogenic [208], [209], [210], secreting high levels of TGF-β [178], [211], strong inflammatory stimuli mature L-DC [178] to preferentially [178] crosspresent [177], [178] MHC I epitopes. This drives high avidity, antigen-specific cytotoxic T cell responses against epidermally acquired antigens [178]; human dDC preferentially stimulate antibody responses [178].

However, in Anopheline mosquito-bites, infiltrating mast cells rapidly degranulate [190], releasing stored TGF-β1 [212] and bioactivating molecules [212], activating TGF-β signalling [213]. Exogenous bioactive TGF-β inhibits maturation and endogenous antigen presentation by immature human L-DC after antigen uptake [212], [214], and upregulates IL-10 secretion in T cells [215] and mDC [216] subsets (this includes L-DC [217]).

Immature human mDC [216], [218], [219], [220], [221], [222] and antigen-activated human pDC [222], [223], [224], (which also secrete chemokines that attract circulating T cells [202]), prime IL-10-secreting, immunosuppressive iTregs from interacting antigen-cognate T cells. Suboptimal [225], or immature DC antigen presentation [218], [219], [226] and low antigen dose [225], [227], [228] preferentially [229] activate Tregs over effectors, and increasing local TGF-β and IL-10 concentrations further activate Tregs [93], [230], [231], [232]. This tolerogenic cascade will act on recruited T cells and Tregs, potentially including malaria-crossreacting specificities [233] present in malaria-naïve people [55], [77], [234].

Once activated, nTregs also secrete bioactive [87] TGF-β1 [87], [235], [236], [237], and most iTregs produce high levels of IL-10 [221], [222], [238], [239] and/or TGF-β [194], [222]. These cytokines mediate multiple Treg activation [87], [216], [221], [231], [240], [241], [242], [243], induction [91], [216], [238], [239], [244], [245], [246], [247] and suppression mechanisms both antigen specific [95], [231] and non-specific [87]. Also, independently of DCs, accumulating human antigen-activated CD4+ Tregs can themselves generate suppressive iTreg via TGF-β-dependent [247], [248], [249] or IL-10 dependent [230], [238] infectious tolerance. These cascading mechanisms will efficiently expand miniscule numbers of cognate Tregs [93], [100], [247] initially activated or induced in location, such as an Anopheline bite-site or draining LN.

#### 4-v. Antigen presentation during mosquito bites provides for opportunistic systemic subversion to malaria- specific tolerance

Critically, Tregs normally home to inflamed tissue [193] and LNs [250] according to homing molecules expressed [251], and in inflamed skin [91], [102], [195] and LNs [252], rapidly out-proliferate [252] and suppress priming [229], [253] and proliferation [252] of same-specificity effector T cells. Under such Treg-dominated [88], [254], or tolerogenic IL-10-rich microenvironments [238], [255], mature human mDC will also generate iTregs [238], [255], [256]. This further facilitates specific systemic tolerization to skin-encountered antigens. High, tolerogenic IL-10 levels normally prevail within 8 hours in LNs draining uninfected Anopheline mosquito bites [190], [257]. The normal response to Anopheline bites therefore, provides a tolerogenically predisposed microenvironment, in which parasites, when transmitted, will drive parasite-specific tolerization.

Evidently, antigen presentation in skin during malaria-infected mosquito bites provides major sources of antigen-specific Tregs capable of repressing local [91], [100], LN and systemic [195], [258] inflammatory reactions to bite-site antigens. T cell repertoires in malaria-naïve people include malaria-cognate specificities [55], [77], [234]. Malaria proteins contain amino acid sequences highly conserved in human housekeeping proteins [233], implying the pre-existence of malaria-cognate nTregs. These T cell and Treg repertoires, responding to mosquito-allergen inflammation, and the minimal malaria antigen doses transmitted in bites, provide amply for *de novo* malaria-specific suppressive iTreg generation, and expansion of pre-existing, potentially cognate [233] nTregs, via Treg induction and expansion mechanisms [225] documented in the basic literature (see above Section *4-iv*). Existing mechanisms for rapidly expanding Treg number [221], [222], [225], [249] and specificity spectrum [93] clearly support rapid induction of antigen-specific Tregs and systemic tolerance [93]. These conditions (part of the normal host response to mosquito bites), occur in malaria infections at earliest skinstage, well before bloodstages emerge. This facilitates opportunistic Treg induction triggered at the skinstage by transmitted parasites. In corroboration, in naïve animals, sand-fly transmitted skinstage *Leishmania* parasites induce *de novo* parasite-specific Tregs (previously undetectable in the animal) [259] which inactivate robust, protective cytotoxic responses [260] concomitantly elicited by parasite infection. Conversely, and significantly, people in endemic areas deficient in basic functions driving Treg induction (TGF-β production, TGF-β receptors, FOXP3, CTLA-4) resist infection [155], not just bloodstage disease, specifically implicating the mechanisms of Treg induction and expansion described, in host susceptibility.

#### 4-vi. CQ specifically blocks Treg-inducing antigen presentation: this prevents malaria-specific tolerance induction via bite-site parasite interactions in the skin

Skin-accumulating CQ disrupts bite-site antigen presentation leading to Treg-induction and activation. Malaria-naïve people treated simultaneously with CQ develop strong immunity via infected bites [163]. A malaria-naïve person therefore, develops protection against natural challenge when rapid cross-presentation of exogenous MHC I, and all MHC II antigen presentation, and consequently also, major sources of rapid antigen-specific activation and *de novo* induction of tolerogenic CD4+ and CD8+ regulatory T cells in the skin and skin-draining LN, are blocked at the skinstage during immunizing skin infections. Slower, protective CD8+ responses to liverstages, also primed in the peripheral lymph nodes [111], [261] are uninhibited. As seen (Section 4-ii), protection due to expression of a broader liverstage antigen repertoire permitted by CQ, as proposed by others [163] is inconsistent with lifelong susceptibility in populations from endemic areas, and other, experimental data. Therefore, live parasite immunization via the skin under CQ immunomodulation, results in protection [163] because CQ coadministered with naturally transmitted parasites, in malaria-naïve people, effects an immunological bypass of rapid, initial skinstage antigen-specific tolerization and suppression, enhancing slower-developing immunity, and memory responses.

#### 4-vii. Enhancing inflammation also counteracts tolerization via the skin

Multiple-bite immunizations deliver unnaturally high allergen/parasite-antigen doses, provoking strongly pro-inflammatory microenvironments. Pro-inflammatory conditions counteract [100], [262], and inhibit [227], [263] DC tolerization and directly disrupt Treg-induction and activation in mucosal skin [264], and can convert Tregs to effectors [265]. Multiple-bite immunizations therefore, support immediate, predominantly cytotoxic [178] malaria-specific protective immune responses, effectively disrupting skinstage tolerization, and enhancing protective memory responses.

### 5. A role for the skin accommodates conflicting data

Consistent with this perspective, genetically attenuated immunizing parasites, (p52/36 [171] or p36 [128]) administered intravenously, entirely bypass skinstage interactions. Multiple short immunizing liverstage infections, of sufficient dosage [171], accumulate enough antigen exposure to establish protective responses. Without inhibitory skinstage interactions, intravenous immunization eliciting swift [46] limited [46], [79] responses to incipient liverstage infection will protect [79], [266] against natural challenge without antigen persistence [171], [267], reconciling these (and other [165], [166], see Supplementary Text S1) contradictory data.

In strong support, multiple cycles of intravenous infection and subsequent elimination with primaquine (PQ), a parasiticidal drug cure, administered during liverstage development (allowing limited antigen presentation by infected hepatocyte [268] before PQ-cure) builds robust protection to natural challenge [129]. Immunization via skin, however, with identical PQ-cure cycles, reduces protection [129] even against intravenous challenge, again implicating the skin in inefficient development of immunity.

Finally, 56% [9] −66% [10], [11] of infants (3–17 and 2–4 months old, respectively) from seasonal [269], high transmission areas can be immunized against natural challenge [9], [10] with a skinstage antigen (CSP) vaccine, that negligibly protects, or does not protect adults from endemic areas [14], [15].

Adult endemic-region populations are largely pre-exposed [270], [271], [272] to malaria parasites in the skin: high-transmission areas provide over 2 infected bites per night [30], [273], [274]. However, neonates are skinstage malaria-naïve. Babies 0–6 months old, enrolled in medical trials, have significant chances of being skinstage-naïve upon immunization, and remaining so for 3–6 months. Urbanizing environments [271], increased bednet use (about 80% compliance [9]) and seasonal transmission relative to birthdate, all reduce exposure to mosquitoes. This allows many intramuscularly immunized infants to pre-establish robust protective responses before encountering skinstage parasites.

### 6. A critical role for immune mechanisms within the skin in malaria vaccine malfunction

Chronic pre-exposure of skin to live parasites coincides closely with failure of clinically functional vaccines (Figure 2A). Immunization reliably protects against natural challenge only in skinstage-naïve individuals where robust responses are established *before* infective parasites ever interact with unmodified skin (Figure 2b,d,g). Available epidemiological and vaccine trial data strongly implicate the skin in a block to protective immunity dependent on the presence of skinstage parasites and functional CD4+ and rapid CD8+ exogenous-antigen presentation in the skin, and independent of prior bloodstage infection. T cell memory responses develop normally during [80], [174], [275] and after [275] bloodstage infection, and human bloodstage-induced immunosuppression [276], [277], [278], [279] is usually limited to acute malaria [279], [280], [281]. The low incidence of acute malaria in semi-immune [32], [33] endemic adults cannot, therefore, account for unmitigated susceptibility to reinfection, nor uniform inability of diverse clinically functional vaccines to protect healthy, endemic adults.

We conclude potentially protective liverstage and vaccine-generated T cell responses, which indisputably exist, are disabled by parasites in the skin.

### 7. Timing, behaviour and molecular characteristics of the malaria parasite skin stage are aggressively tolerogenic

#### 7-i. A role for the bite-site and parasite behaviour in early systemic tolerization

Malaria-infected mosquito bites of 1 minute deposit around 20 [107] sporozoites in nanolitres [152] of saliva into epidermal [107], [110] and dermal [110] skin. Within an hour, in mice, about 10–15% [107] (normally, 2–3) of deposited sporozoites enter proximal skin-draining lymph nodes (LN) [103], [107], [111], rapidly metamorphosing into bloodstage-like forms [107], (we propose the term “pseudomorphs”) expressing antigens [107], [282] characteristic of later liver and bloodstages [282]. After 7 hours [107], most LN parasites are inside [107] or entwined around CD11c+ [107] (cross-presenting) dendritic cells. Some parasites (∼10) remain in the skin [107], and their exact fate is formally unclear. Antigens draining from these will initially reflect the skinstage. However, host cell environment differentially affects expression profile in malaria parasites [283], [284]. Given the metamorphic propensity of LN parasites, antigenic representation of further lifecycle stages in skin-lingering parasites cannot be excluded.

These data are crucial: they reveal the very earliest stages of natural infection immediately expose the host immune system, via skin and rapidly developing “pseudomorphs” in skin-draining LN, to very low doses of (minimally) both skinstage and liverstage antigens. These conditions anad location are highly conducive to antigen-specific Treg induction and activation, and particularly rapid induction of systemic tolerance [285], and therefore, rapid systemic tolerance to parasite antigens arriving in the LN.

#### 7-ii. Normal bite-site responses lead rapidly to systemic tolerance

Tregs activated in skin bite-sites and LN by migrating parasites and draining antigens will rapidly orchestrate systemic tolerance. Tregs tolerize surrounding microenvironments [88], [100], [254], downregulating mDC antigen presentation [286] and upregulating mDC TGF-β and IL-10 secretion [254], [286], [287]. This favours further Treg activation. Tregs also reverse TLR activation of strong proinflammatory responses in human mDC [286], [287], [288], normally triggered by pathogen ligands [289]. Contact with accumulating Tregs induces immature human L-DC and mDC to remain semi-immature [286], [287] and migrate to draining LNs [287]. LN-migrating mDC can transfer peripheral antigen to LN-resident DC [290] such as human pDCs, which congregate in densely packed [291] naïve T cell regions [292]. More immediately, pDC cross-presentation will induce IL-10-secreting Tregs [223], [224] in response to rapidly draining skin-located antigens [291] (eg. from bite-site) and LN-migrating parasite antigens, potentially within an hour of the mosquito bite. Later arriving [293] semi-mature L-DC, trafficking antigen [293] to naive T cell regions of the LN [294], [295] will therefore encounter pre-established, tolerizing microenvironments. Accordingly, skin inflammations [296], Anopheline mosquito-bites [190], [257], and activated TNF-α producing mast cells [199], all increase immature [296] L-DC migration and accumulation in the LN [190], [257], [296]. Also, for isolated Anopheline mosquito bites leukocyte infiltration [190] cross-presenting DCs [190] carrying sporozoite antigen [111] and IL-10 concentration [257] rapidly increase in draining LNs.

These data strongly suggest skinstage parasites capitalize on host responses to isolated, uninfected Anopheline bites, (which are normally immunosuppressive [257]), efficiently misdirecting systemic responses to ensure tolerance to subsequently developing parasites.

#### 7-iii. Skinstage parasite molecules are intrinsically tolerogenic

Malaria parasites also display aggressive molecular intervention strategies. Critically, gliding sporozoites [297] transiting skin cells [298], [299] the LN lumen [107] and invading LN DCs [107], will shed circumsporozoite protein (CSP) and thrombospondin-related-adhesive protein (TRAP) [297], [300]. Cytoplasmic CSP, shed by infecting parasites, inhibits host transcription activator NF-Kβ [301], strongly downregulating multiple pleiotropic pro-inflammatory (anti-parasitic [302]) activities including IL-6 [301] during plasmodial liver infection. It is well established in non-malarial systems that IL-6 averts CD4+ iTreg formation [303], [304], [305], [306] and crucially, suppresses the antigen-specific CD4+ Treg activity [307], [308], which inhibits both T cell activation to foreign antigen [307] and CD4+ and CD8+ T cell memory development [308], [309]. This directly infers IL-6 blockade by malarial CSP [301] prevents immediate protective T cell activation and memory responses in the skin, LN and infected hepatocytes, simultaneously stimulating in these locations antigen-specific Treg formation [303], [304], [305], [306], [310], [311] and expansion [305], triggered by malaria antigens. This will limit any malaria-specific responses arising. Accordingly, immediate *in vivo* T cell responses to malaria-infected hepatocytes [46] are “self-limiting” [79], [266], and plasmodial liver infection, (an extensive tissue insult), is non-inflammatory and asymptomatic [114]. Further, high dose antigen-presentation, which counteracts Treg suppression [227], in the case of malaria-antigens [312] relieves self-limitation of malaria-specific responses [312], implicating malaria-specific Tregs in self-limiting immune responses to infected hepatocytes.

Also, *P. falciparum* TRAP (expressed in both skin and blood stages [313]), like human thrombospondin, bioactivates latent human TGF-β [314] via the TSR-1 domain [315]. Thrombospondin, by a TGF-β-dependent [316] mechanism [212], [239], converts cognate CD4+ T cells into suppressive iTregs *in situ* [316] conferring localized tissue tolerance in mice [316]. In humans, parasite-driven TGF-β bioactivation [314], [317] precedes and correlates strongly with significantly increased Treg numbers and parasitaemia densities [317], and suppresses proinflammatory responses in humans [317] and mice [318]. In unimmunized mice, co-inhibition of TGF-β and IL-10 early [318] in infection, or depletion of Treg [319], restores proinflammatory responses and parasite clearance. Correlative, functional data therefore strongly suggest Treg-inducing skinstage function for TRAP molecules.

#### 7-iv. Skinstage parasite behaviour is potently tolerogenic

Human allergen therapy boosts antigen-specific systemic tolerance by chronic low doses applied via the skin [285], [320], and requires Treg induction and activation [258], [320]. Tolerization is drastically accelerated by low-dose antigen frequently introduced directly into skin-draining LNs [285].

Similarly, people constantly exposed to infected bites inevitably collect frequent low numbers of CSP/TRAP-shedding, LN-migrating, and skin-lingering “pseudomorphic” parasites. This directly infers parasite instigation of continuous Treg activation and induction in both skin and LN, and inevitably, potent intralymphatic, antigen-specific systemic tolerization to exposed malaria antigens.

### 8. A model for skinstage-initiated immune subversion incorporates normal immunobiology of Treg induction and mechanism, and the cadence, behaviour and cellular biology of the parasite lifecycle stages

#### 8-i. The model

Cellular and molecular data indicate isolated malaria-infected mosquito bites drive rapid systemic tolerization to malaria antigens at the skinstage. Since naturally transmitted parasites progress from skin to liver to blood, liver infections inevitably present malaria sporozoite [182], [268] and liverstage antigens to an already efficiently compromised host immune system.

Tregs from human skin are highly proliferative *in vivo*, develop alongside effector responses at the site of a skin inflammation [321], and can be induced *in vivo* and *ex vivo* from highly differentiated memory T cells, by antigen reencounter [321], [322]. Tregs *in vivo* are preferentially [102], [228] induced and activated, and have an *in vivo* proliferative advantage [102], [252], [323] over non-Tregs. These properties provide a clear systemic advantage to any suppressive malaria-specific Tregs generated at the skinstage, over simultaneously elicited malaria-specific [111] protective cytotoxic T cell responses.

Skinstage pre-exposure of later life-cycle antigens [107] provides an additional temporal advantage: liverstage infections subsequently presenting CSP [268] and pre-exposed liverstage epitopes (eg TRAP, EXP-1) will enhance pre-established parasite-protective Treg populations, suppressing swift cytotoxic responses initiated against infected hepatocytes. B cells require activated T cell help to initiate IgG antibody responses, and Tregs will also directly, antigen-specifically and non-specifically, inhibit or kill activated B cells [89], [90], [92], [324]. Antibody responses and B cell function will therefore also be vulnerable to repression by skin-induced, malaria-specific Tregs, from the skinstage onwards. Infection, and reinfection, not sterile immunity, will prevail in natural transmission.

Induction of broad-spectrum malaria-specific Tregs in the skin therefore, ensures liverstage infections always develop into transmissible blood stages. At least 281 bloodstage proteins are expressed at the skin stage, including AMA-1, PfEMP and STEVOR proteins [325]. More than one liver- and bloodstage-expressed antigen (eg TRAP, EXP-1) [313], [326], [327] is definitely exposed at the skinstage [107], [313], and therefore to the skin-based tolerizing mechanisms we define. *Once Tregs are induced, numerous mechanisms (see Sections 4-iv, 4-v, 7-iii) exist allowing small numbers of Tregs of one specificity to expand, outgrow, convert non-Tregs of different specificities to suppressive function (infectious tolerance), and also non-specifically suppress responses to other antigens (bystander suppression)* [*93*] *.* Therefore, a Treg response specific and suppressive to one liverstage or bloodstage antigen (eg. TRAP or EXP-1), triggered initially during skinstage, can later re-expand upon specific antigen re-encounter at the liver or bloodstage, and non-specifically suppress T cell (or ablate memory B cell) responses to further parasite antigens co-expressed only at the later stage, eg. responses to MSP-1, specific to late-liver and bloodstages. Pre-induction of bloodstage-specific Tregs provides an obvious protective advantage promoting the establishment of bloodstage parasites emerging from the liver. Re-expansion of pre-induced Tregs will initiate a tolerogenic cascade and immunosuppressive environment conducive to bloodstage expansion, which is further enhanced by the bloodstage parasite themselves. Bloodstage parasites generate Treg activity [317], [319], [328], [329] and nonspecific immunosuppressive conditions [85], [314], [329], [330], [331], [332], enhancing systemic tolerance [328], [333] conducive to multiple parasitaemia cycles, thereby increasing probability of gametocyte transmission, and also reinfection [85], [334].

#### 8-ii. Model outcomes: experimental versus field infections

**Figure 3:**
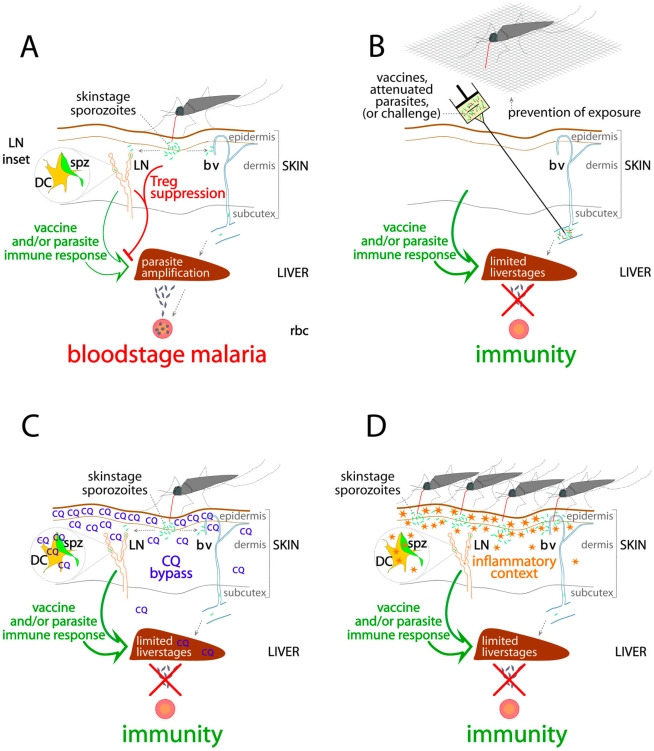
All protective immunization circumvents initial malaria-specific Treg activation in the skin: a functional model. A: Natural transmission (mosquito bite) allows skinstage parasites (sporozoites, spz, green fragments) to migrate (dotted gray arrows) through skin cells to both lymph nodes (LN) and liver (via blood vessels, bv), and induce malaria-specific regulatory T cells in the skin and LN that suppress (red blocker lines) local and systemic protective immune responses (green arrows), resulting in bloodstage infections (gray fragments below liver), and amplification cycles within red blood cells (rbc). B, C, D: Protective immunity develops where immunization and/or challenge avoids parasite-skin interaction and Treg activation/induction; B: Physical bypass of the skin, (by intravenous (i.v.) attenuated parasites, purified antigen, antibody or intramuscular or intranasal subunit vaccine) at immunization, or i.v. challenge with unattenuated infective parasites, avoids all induction or activation of skin and liverstage-specific Tregs. C: Chloroquine (CQ) accumulates and blocks all MHC II and rapid, (but not classical, slow) MHC I malaria-antigen presentation in the skin, which would otherwise immediately induce/activate antigen-specific Tregs. D: Multiple simultaneous mosquito bites create a strongly pro-inflammatory local skin and/or systemic milieu which inhibit Treg activation and induction processes. LN Inset: Inside LN, metamorphosing skinstage sporozoite parasites (spz) are in close contact with, or invade, host antigen presenting dendritic cells (DC).

Experimental, intravenous sporozoite infections bypass skin/LN Treg activation (Figure 3B). Consequently, predominant immune responses to liverstage infection, which prime in the peripheral LN [111], [261] are unhindered, rendering the immunodominant CSP molecule and also TRAP, experimentally protective [335], [336], [337].

Like physical bypass of immune interactions in the skin at immunization or challenge (Fig. 3B), immunological bypass by CQ inhibition of antigen presentation (Fig. 3C), or inflammatory inhibition of tolerizing cellular cascades (Fig. 3D) during immunization, consistently result in protection to natural skinstage challenge for malaria-naïve subjects.

Natural, skin-initiated infections however, pre-establish antigen-specific tolerance to parasite antigens presented in the skin and LN (Fig. 3A). In fundamental opposition to current opinion [338] and vaccine dogma, this model shows CSP and TRAP are pleiopotently immunosuppressive molecules when deployed within the cadence of natural infection. Other lifecycle-stage antigens pre-exposed by skinstage “pseudomorphs” [107], such as liver and bloodstage antigens, broaden the scope of immunosuppressive systemic responses to parasite antigens subsequently encountered during liverstage or bloodstage infection (or immunization). Critically, again directly opposing prevailing opinion, further exposure to infected mosquitoes will boost tolerance, and suppression of protective responses, not immunity.

#### 8-iii. Selective vaccine failure: who, where and why

Adults chronically exposed to infected mosquitoes from birth will accumulate broad repertoires of regulatory T cells, becoming potently pretolerized. Vaccination deploying skinstage-exposed or bloodstage antigens will activate both pre-existing cognate Tregs [225], [252], [339] as shown for foreign antigen in mouse systems, and malaria-specific T cells, (effector and other subsets) as shown in humans [340], [341]; challenge by mosquito bite will preferentially boost pre-existing Treg-based tolerance [225], [339], suppressing protective concomitantly elicited and recall responses [339]. Vaccines which substantially protect malaria-naïve adults [12], [13] will negligibly, or not, protect endemic-area adults [14], [15], as solidly evidenced by adult vaccine field trials.

Neonates, initially skinstage-malaria-naïve, are easier to protect from mosquito bite. Accumulation of skinstage-induced regulatory T cells specific for malaria will be negligible, and immunization will (potentially unrestrictedly) favour [69] immunity. Endemic-area infants, like malaria-naïve adults, should be better protected by formulations that negligibly protect endemic-area adults. This profile is precisely corroborated by results for leading CSP-containing RTS,S, vaccine formulations. These protect malaria-naïve adults partially [12], [13], endemic-area infants (2–17 month-old) similarly partially [9], [10], [11], older children poorly [7], [8], [342] and endemic-area adults negligibly [1], [14], [15].

## Discussion

### Limitations of the study

Using rigorous systematic literature search, and individual screening with unambiguous criteria on nearly 2000 studies, we have identified the great majority of peer-reviewed, experimental evidence documenting complete protection to malaria infection. Meta-analysis of experimental conditions involved shows only immunizations that avoid live parasite interaction in the skin, or inhibit regulatory T cell induction within the skin during skin-immunization, fully protect against infected mosquito bites. Our main conclusion, that very early, skinstage-induced, antigen-specific regulatory T cells block malaria vaccine function therefore rests empirically but solidly on a straightforward meta-analysis of unbiased experimental data. The data were retrieved from experiments carried out worldwide, by hundreds of groups, using many different protocols over many decades, and drawn from extensive literature reporting skinstage, liverstage and bloodstage vaccine trials. This minimizes both experimental bias and the impact inherent in overlooking any one published study, but does not specify a mechanism. However, detailed molecular/cellular immunological data from experiments carried out independently, in the main by basic research groups unconcerned with malaria (and therefore unbiased), define a mechanism of tolerogenic induction via the skin which strongly supports this conclusion. Combined with the cadence of the parasite lifecycle stages, and the behaviour and detailed cellular and molecular character of skinstage parasites during transmission, we define a novel immuno-epidemiological model for vaccine success/failure. This identifies differential exposure to infected mosquitos and malaria-specific Treg accumulation as the basis for selective vaccine failure in endemic-area populations, and defines a skin-triggered immunological mechanism for early tolerance induction, preceding and independent of later bloodstage immunosuppression. The available epidemiological and experimental vaccine trial data are entirely consistent with the immunological mechanism and model we propose. The model specified by these combined data easily explains myriad long-standing incongruities, paradoxes and contradictions in the malaria literature. In 1916 studies, we could find no experimental immunization or natural infection data which contradict or weaken our conclusions.

### Fundamental implications for vaccine efficacy

Circumvention of malaria-specific regulatory T cell activation in the skin fits all identified instances of protective immunization against virulent experimental challenge, and selective protection in endemic skinstage challenge. Fundamental immediate and longterm implications for vaccine development and strategy are highlighted below.

#### i. Live parasite vaccines

Unmodified intradermal delivery [148] will obliterate efficacy in otherwise outstandingly successful attenuated-parasite vaccines, advocating optimization of immune context for dermal delivery.

#### ii. Antigen selection

Intrinsically tolerogenic antigens (eg. CSP, TRAP), or those enriching skinstage and/or bloodstage-cognate Tregs (eg. TRAP, EXP-1), (or abundant bloodstream antigens, eg. AMA-1, MSP-1, which will almost inevitably induce homeostatic regulatory T cell activity), are counter-productive in pre-tolerized individuals, although initially effective in malaria-naïve individuals, such as neonates. With age, increasing bite exposure and skin-induced malaria-specific Treg accumulation will repress T cell and B cell responses, eroding [90], [92] skinstage-specific, antibody-correlated [10], [343], [344], [345] infant protection elicited by leading vaccines such as RTS,S. Of particular concern, vaccination with intrinsically tolerogenic antigens although initially protective in malaria-naïve infants, will also predispose bite-exposed growing children to severe malaria, increasing individual risk of later childhood death. Late-midliverstage antigens (unexposed at skinstage) however, should initially escape significant Treg induction and memory inhibition in the skinstage, and do show protectivity [17], [26], [27], [68], [346], [347] into adolescence [347]. This makes late-midliverstage antigens rationally preferred candidates for more universally applicable subunit vaccines with less hindered immune memory.

#### iii. Infant mortality

Neonates protected from mosquitoes will not accumulate skinstage-specific Tregs. Immunization under Treg pre-emptive conditions from birth (non-tolerogenic antigen, CQ accumulation in the skin, scrupulous use of bed-nets/insect-repellent), inducing abundant skinstage-specific antibodies should substantially reduce infant infections beyond currently obtainable protection. Additional early-infancy immunization with liverstage [347] and bloodstage antigens [29], [30] will rapidly [29], [30], [33], [348], [349] provide robust [29], [30] bloodstage immunity. This provides critical protection against increased risk of severe malaria [274] due to decreased exposure to bloodstage disease. Substantially reduced childhood morbidity and mortality, and shrinking transmission reservoirs, should result.

#### iv. Eradication

Discarded vaccines, ineffective in endemic adults but pretested for safety, immunogenicity and tolerability can be rapidly retested (given voluntary patent rescindication [350]) for protectivity in neonates under Treg pre-emptive conditions. Bednets and CQ are cheap, CQ is usually well-tolerated, including during pregnancy [158], [351], and is transmitted transplacentally at therapeutic doses and via breast milk at subtherapeutic dosages [351]. CQ accumulates preferentially in the skin, so significantly reduced, subtherapeutic dosage may suffice. Treg-blocking (immunomodulary) pharmacological effects of CQ are on the host, not parasite, eliminating parasite-resistance obstacles. Current infrastructure (http://www.theglobalfund.org) allows largescale immunization across (primarily African) endemic regions worldwide.

Malaria outside Africa is mostly hypoendemic [270], and not all Africa is holoendemic [270]. With increasing bednet/insecticide use, transmission drops [270], [272], [352], [353]. Coordinated neonate vaccination generating additional concerted widespread reductions (herd effects) in infectious reservoirs is feasible, even with low efficacy vaccines [354]. Conditions pre-empting Treg induction at early skin stage will amplify trends leading to interrupted transmission, catalyzing [272] significantly accelerated local elimination, and facilitating worldwide eradication of malaria.

Existing adult transmission reservoirs however, will counteract shrinking infant reservoirs [272], [354]. Skinstage-induced-Treg evasion and memory enhancing vaccines, preventing adult re-infection, and transmission-blocking vaccines targeting parasite development within the mosquito, and antimalarials blocking bloodstage transmission, are therefore essential to fast-track eradication.

### Wider perspective

Skinstage activation of parasite-specific Treg-based systemic immunosuppression provides a fundamentally new, experimentally widely substantiated, immunological rationale, and precise focus, for research and vaccination strategy leading to potentially accelerated malaria eradication. The concept, and implications for vaccination, apply to closely related and economically important (*Toxoplasma, Theileria*, *Babesia* spp.) pathogens.

## Supporting Information

Table S1 Complete protection data (177 experiments) reference list for meta-analysis.
(0.24 MB DOC)

Table S2 Protective vaccination physically bypasses the skin at immunization or challenge (90%) or involves skin immunomodulation (10%). A. Exposure to parasites in the skin coincides closely with vaccine failure. Green background- immunization procedures. Lilac background- challenge procedures and percent of total experiments showing complete protection (% total) formed by a subset of studies (category) using a given experimental procedure (categories a-i; supporting experimental data for each category is in references listed below; also Supplementary Table S1). Parasite immunization administration routes: (a,b): live parasites given intravenously (i.v.), or the method does not involve live parasites, but uses dead parasites or purified antigen, antibody, or recombinant DNA (subunit) and therefore bypasses parasite interactions with host skin; (c,d,e,f,g,h): live parasites are administered via the skin; (c,d): by multiple simultaneous mosquito bites/session; (e,f): live parasites are naturally transmitted by 4–15 bites; (g): live parasites are administered by 12–15 bites/session with chloroquine; (h): live parasites delivered subcutaneously (s.c) or intradermally (i.d.) or intramuscularly (i.m.); (i): uncontrolled exposure to endemic mosquitos. Challenge route is either i.v. or by mosquito bite, as indicated. B. Protective immunization bypasses the skin at either immunization or challenge in 90% of cases: categories (a,b,c,e,h) shaded blue with red crosses. Protective immunization which transits skin during immunization (c,d,e,f,g,h) either: bypasses the skin physically at challenge (c,e,h) (red cross); or, involves skin immunomodulation during immunization (d,g, 10% of cases). Asterisk (*)- immunization via unmodified skin, limited to P. berghei (f). Skin bypass (red cross)- method physically avoids live parasite interactions in the host skin. Malaria exposure- skin exposure to infected mosquito bite before first immunization; naïve- no pre-exposure; exposed (endemic)- chronic exposure. x- this study shows complete protection of 40 of 41 mice challenged. y: one person in one study [117] was infected one time via the skin prior to protective immunization and was therefore moderately tolerized. Data pertaining to experimental categories (a–h): a:[35], [42], [47], [49], [50], [51], [57], [68], [70], [71], [72], [73], [74], [75], [76], [78], [85], [124], [125], [126], [128], [129], [130], [133], [137], [138], [139], [150], [151], [166], [169], [171], [173], [185], [267], [335], [336], [337], [346] [355], [356], [357], [358], [359], [360], [361], [362], [363], [364], [365], [366], [367], [368], [369], [370], [371], [372], [373], [374], [375], [376], [377], [378], [379], [380], [381], [382], [383], [384], [385], [386] [387], [388], [389], [390], [391], [392], [393], [394], [395], [396], [397], [398], [399], [400], [401], [402], [403], [404], [405], [406], [407], [408], [409] [410], [411], [412], [413], [414], [415], [416], [417], [418], [419], [420], [421], [422], [423], [424], [425], [426], [427], [428], [429], [430], [431], [432], [433], [434], [435], [436], [437], [438], [439], [440], [441], [442], [443], [444], [445], [446], [447], [448], [449], [450], [451]. b: [47], [48], [124], [128], [129], [138], [171], [387], [452], [453]. c: [124]. d: [36], [44], [118], [119], [120], [121], [122], [123], [124], [134], [135], [136], [172], [454], [455], [456]. e: [111], [132], [457]. f: [127]. g: [163]. h: [48], [111], [388], [458], [459]. Studies containing data for multiple relevant experimental conditions are referenced accordingly in each appropriate category. Multiple experiments contributed by a single study are indicated beneath study reference number (eg. reference 124 X2) in Supplementary Table S1. (Meta-analysis data extended reference list).
(8.70 MB TIF)

Text S1 Antigen persistence in liverstages is not required for protection. Directly contradictory data [165], [166], [171], [267] is also easily reconciled. Like increasing irradiation [149], [165], the drug primaquine (PQ) eliminates liverstages [47], [166]. PQ however, also disrupts membrane and vesicular trafficking [159], [268], temporarily eliminating all antigen presentation. This prevents immunity if used during immunization [166], creating an apparent correlation between protection and parasite persistence [166]. Used after intravenous immunization [129], allowing early liverstage antigen presentation, however, multiple PQ-cure cycles provide sufficient cumulative antigen presentation to build immunity, without antigen persistence.
(0.07 MB DOC)
