# Supplementary material for: Why Functional Pre-Erythrocytic and Bloodstage Malaria Vaccines Fail: A Meta-Analysis of Fully Protective Immunizations and Novel Immunological Model
Source: PLoS One. 2010 May 19;5(5):e10685. doi: 10.1371/journal.pone.0010685 (PMC2873430; doi:10.1371/journal.pone.0010685)
Supplement: Table S1 — Complete protection data (177 experiments) reference list for meta-analysis. (0.24 MB DOC) [file pone.0010685.s001.doc]

**Supplementary Table S1: Complete protection data (**177 experiments) reference list for meta-analysis

| Category | Ref.nr | Publication |
| --- | --- | --- |
| **a** | 35 | Romero P, Maryanski JL, Corradin G, Nussenzweig RS, Nussenzweig V, et al. (1989) Cloned cytotoxic T cells recognize an epitope in the circumsporozoite protein and protect against malaria. Nature 341: 323-325. |
|  | 42 | Rodrigues M, Nussenzweig RS, Zavala F (1993) The relative contribution of antibodies, CD4+ and CD8+ T cells to sporozoite-induced protection against malaria. Immunology 80: 1-5. |
|  | 47 | Belnoue E, Costa FT, Frankenberg T, Vigario AM, Voza T, et al. (2004) Protective T cell  immunity against malaria liver stage after vaccination with live sporozoites under chloroquine treatment. J Immunol 172: 2487-2495. |
|  | 49 | Jobe O, Lumsden J, Mueller AK, Williams J, Silva-Rivera H, et al. (2007) Genetically attenuated Plasmodium berghei liver stages induce sterile protracted protection that is mediated by major histocompatibility complex Class I-dependent interferon-gamma-producing CD8+ T cells. J Infect Dis 196: 599-607. |
|  | 50 | Purcell LA, Wong KA, Yanow SK, Lee M, Spithill TW, et al. (2008) Chemically attenuated Plasmodium sporozoites induce specific immune responses, sterile immunity and cross-protection against heterologous challenge. Vaccine 26: 4880-4884. |
|  | 51 | Oliveira GA, Kumar KA, Calvo-Calle JM, Othoro C, Altszuler D, et al. (2008) Class II restricted protective immunity induced by malaria sporozoites. Infect Immun 76:1200-1206. |
|  | 57 | Tartz S, Russmann H, Kamanova J, Sebo P, Sturm A, et al. (2008) Complete protection against P. berghei malaria upon heterologous prime/boost immunization against circumsporozoite protein employing Salmonella type III secretion system and Bordetella adenylate cyclase toxoid. Vaccine 26: 5935-5943. |
|  | 68 | Daubersies P, Ollomo B, Sauzet JP, Brahimi K, Perlaza BL, et al. (2008) Genetic immunisation by liver stage antigen 3 protects chimpanzees against malaria despite low immune responses. PLoS One 3: e2659. |
|  | 70 | Chen DH, Tigelaar RE, Weinbaum FI (1977) Immunity to sporozoite-induced malaria infection in mice. I. The effect of immunization of T and B cell-deficient mice. J Immunol 118: 1322-1327. |
|  | 71 | Spitalny GL, Verhave JP, Meuwissen JH, Nussenzweig RS (1977) Plasmodium berghei: T cell dependence of sporozoite-induced immunity in rodents. Exp Parasitol 42: 73-81. |
|  | 72 | Schofield L, Villaquiran J, Ferreira A, Schellekens H, Nussenzweig R, et al. (1987) Gamma interferon, CD8+ T cells and antibodies required for immunity to malaria sporozoites. Nature 330: 664-666. |
|  | 73 | Weiss WR, Sedegah M, Beaudoin RL, Miller LH, Good MF (1988) CD8+ T cells (cytotoxic/suppressors) are required for protection in mice immunized with malaria sporozoites. Proc Natl Acad Sci U S A 85: 573-576. |
|  | 74 | Tsuji M, Romero P, Nussenzweig RS, Zavala F (1990) CD4+ cytolytic T cell clone confers protection against murine malaria. J Exp Med 172: 1353-1357. |
|  | 75 | Doolan DL, Hoffman SL (2000) The complexity of protective immunity against liverstage  malaria. J Immunol 165: 1453-1462. |
|  | 76 | Belnoue E, Voza T, Costa FT, Gruner AC, Mauduit M, et al. (2008) Vaccination with live  Plasmodium yoelii blood stage parasites under chloroquine cover induces cross-stage immunity against malaria liver stage. J Immunol 181: 8552-8558. |
|  | 78 | Beaudoin RL, Strome CP, Mitchell F, Tubergen TA (1977) Plasmodium berghei: immunization of mice against the ANKA strain using the unaltered sporozoite as an antigen. Exp Parasitol 42: 1-5. |
|  | 85 | Orjih AU (1985) Acute malaria prolongs susceptibility of mice to Plasmodium berghei sporozoite infection. Clin Exp Immunol 61: 67-71. |
|  | 124  x2 | Vaughan JA, Scheller LF, Wirtz RA, Azad AF (1999) Infectivity of Plasmodium berghei  sporozoites delivered by intravenous inoculation versus mosquito bite: implications for sporozoite vaccine trials. Infect Immun 67: 4285-4289. |
|  | 125 | Spitalny GL, S. NR (1972) Effect of various routes of immunization and methods of parasite attenuation on the development of protection against sporozoite-induced rodent malaria. Proc Helm Soc Wash 39: 506-514. |
|  | 126 | Kramer LD, Vanderberg JP (1975) Intramuscular immunization of mice with irradiated Plasmodium berghei sporozoites. Enhancement of protection with albumin. Am J Trop Med Hyg 24: 913-916. |
|  | 128 | Douradinha B, van Dijk MR, Ataide R, van Gemert GJ, Thompson J, et al. (2007) Genetically attenuated P36p-deficient Plasmodium berghei sporozoites confer long-lasting and partial cross-species protection. Int J Parasitol 37: 1511-1519. |
|  | 129 | Putrianti ED, Silvie O, Kordes M, Borrmann S, Matuschewski K (2009) Vaccine-like immunity against malaria by repeated causal-prophylactic treatment of liver-stage Plasmodium parasites. J Infect Dis 199: 899-903. |
|  | 130 | Sedagah M, Weiss WR, Hoffman SL (2007) Cross-protection between attenuated Plasmodium berghei and P. yoelii sporozoites. Parasite Immunology 29: 559-565. |
|  | 133 | Collins WE, Skinner JC, Millet P, Broderson JR, Filipski VK, et al. (1992) Reinforcement of immunity in Saimiri monkeys following immunization with irradiated sporozoites of Plasmodium vivax. Am J Trop Med Hyg 46: 327-334. |
|  | 137 | Fandeur T, Gysin J, mercereau-Puijalon (1992) Protection of squirrel monkeys against virulent Plasmodium falciparum infections by use of attenuated parasites. Infect Immun 60:4:1390-1396. |
|  | 138 | Ting LM, Gissot M, Coppi A, Sinnis P, Kim K (2008) Attenuated Plasmodium yoelii lacking purine nucleoside phosphorylase confer protective immunity. Nat Med 14: 954-958. |
|  | 139 | Hirunpetcharat C, Tian JH, Kaslow DC, van Rooijen N, Kumar S, et al. (1997) Complete  protective immunity induced in mice by immunization with the 19-kilodalton carboxyl-terminal fragment of the merozoite surface protein-1 (MSP1[19]) of Plasmodium yoelii expressed in Saccharomyces cerevisiae: correlation of protection with antigen-specific antibody titer, but not with effector CD4+ T cells. J Immunol 159: 3400-3411. |
|  | 150 | Puri SK, Maheshwari RK, Dutta GP, Friedman RM, Dhar MM (1988) Human interferon-gamma protects rhesus monkeys against sporozoite-induced Plasmodium cynomolgi malaria infection. J Interferon Res 8: 201-206. |
|  | 151 | Romero JF, Ibrahim GH, Renggli J, Himmelrich H, Graber P, et al. (2007) IL-12p40-independent induction of protective immunity upon multiple Plasmodium berghei irradiated sporozoite immunizations. Parasite Immunol 29: 541-548. |
|  | 166 | Mueller AK, Deckert M, Heiss K, Goetz K, Matuschewski K, et al. (2007) Genetically attenuated Plasmodium berghei liver stages persist and elicit sterile protection primarily via CD8 T cells. Am J Pathol 171: 107-115. |
|  | 169 | Mellouk S, Lunel F, Sedegah M, Beaudoin RL, Druilhe P (1990) Protection against malaria induced by irradiated sporozoites. Lancet 335: 721. |
|  | 171 | Labaied M, Harupa A, Dumpit RF, Coppens I, Mikolajczak SA, et al. (2007) Plasmodium yoelii sporozoites with simultaneous deletion of P52 and P36 are completely attenuated and confer sterile immunity against infection. Infect Immun 75: 3758-3768. |
|  | 173 | Orjih AU, Nussenzweig RS (1979) Plasmodium berghei: suppression of antibody response to sporozoite stage by acute blood stage infection. Clin Exp Immunol 38: 1-8. |
|  | 185 | White KL, Snyder HL, Krzych U (1996) MHC class I-dependent presentation of exoerythrocytic antigens to CD8+ T lymphocytes is required for protective immunity against Plasmodium berghei. J Immunol 156: 3374-3381. |
|  | 267 | Tarun AS, Dumpit RF, Camargo N, Labaied M, Liu P, et al. (2007) Protracted sterile protection with Plasmodium yoelii pre-erythrocytic genetically attenuated parasite malaria vaccines is independent of significant liver-stage persistence and is mediated by CD8+ T cells. J Infect Dis 196: 608-616 |
|  | 335 | Kumar KA, Sano G, Boscardin S, Nussenzweig RS, Nussenzweig MC, et al. (2006) The  circumsporozoite protein is an immunodominant protective antigen in irradiated sporozoites. Nature 444: 937-940. |
|  | 336 | Khusmith S, Charoenvit Y, Kumar S, Sedegah M, Beaudoin RL, et al. (1991) Protection against malaria by vaccination with sporozoite surface protein 2 plus CS protein. Science 252: 715-718. |
|  | 337 | Kumar KA, Baxter P, Tarun AS, Kappe SH, Nussenzweig V (2009) Conserved protective mechanisms in radiation and genetically attenuated uis3(-) and uis4(-) Plasmodium sporozoites. PLoS One 4: e4480 |
|  | 346 | Daubersies P, Thomas AW, Millet P, Brahimi K, Langermans JA, et al. (2000) Protection against Plasmodium falciparum malaria in chimpanzees by immunization with the conserved preerythrocytic liver-stage antigen 3. Nat Med 6: 1258-1263. |
|  | 355 | Martinez PA, Yandar N, Lesmes LP, Forero M, Perez-Leal O, et al. (2009) Passive transfer of Plasmodium falciparum MSP-2 pseudopeptide-induced antibodies efficiently controlled parasitemia in Plasmodium berghei-infected mice. Peptides 30: 330-342. |
|  | 356 | Amante FH, Good MF (1997) Prolonged Th1-like response generated by a Plasmodium yoelii-specific T cell clone allows complete clearance of infection in reconstituted mice. Parasite Immunol 19: 111-126. |
|  | 357 | Brahimi K, Badell E, Sauzet JP, BenMohamed L, Daubersies P, et al. (2001) Human antibodies against Plasmodium falciparum liver-stage antigen 3 cross-react with Plasmodium yoelii preerythrocytic-stage epitopes and inhibit sporozoite invasion in vitro and in vivo. Infect Immun 69: 3845-3852. |
|  | 358 | Bruna-Romero O, Gonzalez-Aseguinolaza G, Hafalla JC, Tsuji M, Nussenzweig RS (2001) Complete, long-lasting protection against malaria of mice primed and boosted with two distinct viral vectors expressing the same plasmodial antigen. Proc Natl Acad Sci U S A 98: 11491-11496. |
|  | 359 | Burns JM, Jr., Flaherty PR, Nanavati P, Weidanz WP (2004) Protection against Plasmodium chabaudi malaria induced by immunization with apical membrane antigen 1 and merozoite surface protein 1 in the absence of gamma interferon or interleukin-4. Infect Immun 72: 5605-5612. |
|  | 360 | Cabrera EJ, Barr ML, Silverman PH (1977) Long-term studies on rhesus monkeys (Macaca mulatta) immunized against Plasmodium knowlesi. Infect Immun 15: 461- 465. |
|  | 361 | Cao Y, Zhang D, Pan W (2009) Construction of transgenic Plasmodium berghei as a model for evaluation of blood-stage vaccine candidate of Plasmodium falciparum chimeric protein 2.9. PLoS One 4: e6894 |
|  | 362 | Charoenvit Y, Brice GT, Bacon D, Majam V, Williams J, et al. (2004) A small peptide (CEL-1000) derived from the beta-chain of the human major histocompatibility complex class II molecule induces complete protection against malaria in an antigenindependent manner. Antimicrob Agents Chemother 48: 2455-2463. |
|  | 363 | Charoenvit Y, Collins WE, Jones TR, Millet P, Yuan L, et al. (1991) Inability of malaria  vaccine to induce antibodies to a protective epitope within its sequence. Science 251: 668-671. |
|  | 364 | Charoenvit Y, Majam VF, Corradin G, Sacci JB, Jr., Wang R, et al. (1999) CD4(+) Tcell- and gamma interferon-dependent protection against murine malaria by immunization with linear synthetic peptides from a Plasmodium yoelii 17-kilodalton hepatocyte erythrocyte protein. Infect Immun 67: 5604-5614. |
|  | 365 | Charoenvit Y, Sedegah M, Yuan LF, Gross M, Cole C, et al. (1990) Active and passive immunization against Plasmodium yoelii sporozoites. Bull World Health Organ 68 Suppl: 26-32. |
|  | 366 | Chatterjee S, Druilhe P, Wery M (1999) Irradiated sporozoites prime mice to produce high antibody titres upon viable Plasmodium berghei sporozoite challenge, which act upon liver-stage development. Parasitology 118: 219-225. |
|  | 367 | Chatterjee S, Francois G, Druilhe P, Timperman G, Wery M (1996) Immunity to Plasmodium berghei exoerythrocytic forms derived from irradiated sporozoites. Parasitol Res 82: 297-303. |
|  | 368 | Chatterjee S, Ngonseu E, Van Overmeir C, Correwyn A, Druilhe P, et al. (2001) Rodent malaria in the natural host--irradiated sporozoites of Plasmodium berghei induce liverstage  specific immune responses in the natural host Grammomys surdaster and protect immunized Grammomys against P. berghei sporozoite challenge. Afr J Med Med Sci 30 Suppl: 25-33. |
|  | 369 | Chattopadhyay R, Conteh S, Li M, James ER, Epstein JE, et al. (2009) The Effects of radiation on the safety and protective efficacy of an attenuated Plasmodium yoelii sporozoite malaria vaccine. Vaccine 27: 3675-3680. |
|  | 370 | Clark IA, Allison AC, Cox FE (1976) Protection of mice against Babesia and Plasmodium with BCG. Nature 259: 309-311. |
|  | 371 | Clark IA, Cox FE, Allison AC (1977) Protection of mice against Babesia spp. And Plasmodium spp. with killed Corynebacterium parvum. Parasitology 74: 9-18. |
|  | 372 | Daly TM, Long CA (1995) Humoral response to a carboxyl-terminal region of the merozoite surface protein-1 plays a predominant role in controlling blood-stage infection in rodent malaria. J Immunol 155: 236-243. |
|  | 373 | Degano P, Schneider J, Hannan CM, Gilbert SC, Hill AV (1999) Gene gun intradermal DNA immunization followed by boosting with modified vaccinia virus Ankara: enhanced CD8+ T cell immunogenicity and protective efficacy in the influenza and malaria models. Vaccine 18: 623-632. |
|  | 374 | Doolan DL, Hoffman SL (1999) IL-12 and NK cells are required for antigen-specific adaptive immunity against malaria initiated by CD8+ T cells in the Plasmodium yoelii model. J Immunol 163: 884-892. |
|  | 375 | Egan JE, Weber JL, Ballou WR, Hollingdale MR, Majarian WR, et al. (1987) Efficacy of murine malaria sporozoite vaccines: implications for human vaccine development. Science 236: 453-456. |
|  | 376 | Gilbert SC, Schneider J, Plebanski M, Hannan CM, Blanchard TJ, et al. (1999) Ty viruslike  particles, DNA vaccines and Modified Vaccinia Virus Ankara; comparisons and combinations. Biol Chem 380: 299-303. |
|  | 377 | Gramzinski RA, Doolan DL, Sedegah M, Davis HL, Krieg AM, et al. (2001) Interleukin-  12- and gamma interferon-dependent protection against malaria conferred by CpG oligodeoxynucleotide in mice. Infect Immun 69: 1643-1649. |
|  | 378 | Gruner AC, Mauduit M, Tewari R, Romero JF, Depinay N, et al. (2007) Sterile protection against malaria is independent of immune responses to the circumsporozoite protein. PLoS One 2: e1371. |
|  | 379 | Guebre-Xabier M, Schwenk R, Krzych U (1999) Memory phenotype CD8(+) T cells persist in livers of mice protected against malaria by immunization with attenuated Plasmodium berghei sporozoites. Eur J Immunol 29: 3978-3986. |
|  | 380 | Hirunpetcharat C, Vukovic P, Liu XQ, Kaslow DC, Miller LH, et al. (1999) Absolute requirement for an active immune response involving B cells and Th cells in immunity to Plasmodium yoelii passively acquired with antibodies to the 19-kDa carboxylterminal fragment of merozoite surface protein-1. J Immunol 162: 7309-7314. |
|  | 381 | Hirunpetcharat C, Wipasa J, Sakkhachornphop S, Nitkumhan T, Zheng YZ, et al. (2003) CpG oligodeoxynucleotide enhances immunity against blood-stage malaria infection in mice parenterally immunized with a yeast-expressed 19 kDa carboxyl-terminal fragment of Plasmodium yoelii merozoite surface protein-1 (MSP1(19)) formulated in oil-based Montanides. Vaccine 21: 2923-2932. |
|  | 382 | Hoffman SL, Berzofsky JA, Isenbarger D, Zeltser E, Majarian WR, et al. (1989) Immune  response gene regulation of immunity to Plasmodium berghei sporozoites and circumsporozoite protein vaccines. Overcoming genetic restriction with whole organism and subunit vaccines. J Immunol 142: 3581-3584. |
|  | 383 | Hoffman SL, Crutcher JM, Puri SK, Ansari AA, Villinger F, et al. (1997) Sterile protection of monkeys against malaria after administration of interleukin-12. Nat Med 3: 80-83. |
|  | 384 | Hunter RL, Kidd MR, Olsen MR, Patterson PS, Lal AA (1995) Induction of long-lasting immunity to Plasmodium yoelii malaria with whole blood-stage antigens and copolymer adjuvants. J Immunol 154: 1762-1769. |
|  | 385 | Imai T, Shen J, Chou B, Duan X, Tu L, et al. (2010) Involvement of CD8(+) T cells in protective immunity against murine blood-stage infection with Plasmodium yoelii 17XL strain. Eur J Immunol. In press DOI: 10.1002/eji.200939525 |
|  | 386 | Jaffe RI, Lowell GH, Gordon DM (1990) Differences in susceptibility among mouse strains to infection with Plasmodium berghei (ANKA clone) sporozoites and its relationship to protection by gamma-irradiated sporozoites. Am J Trop Med Hyg 42: 309-313. |
|  | 387 | Aly AS, Mikolajczak SA, Rivera HS, Camargo N, Jacobs-Lorena V, et al. (2008) Targeted deletion of SAP1 abolishes the expression of infectivity factors necessary for successful malaria parasite liver infection. Mol Microbiol 69: 152-163. |
|  | 388 | Aly AS, Downie MJ, Mamoun CB, Kappe SH (2010) Subpatent infection with Nucleoside Transporter 1-deficient Plasmodium blood stage parasites confers sterile protection against lethal malaria in mice. Cell Microbiol. Epub ahead of print |
|  | 389 | Draper SJ, Goodman AL, Biswas S, Forbes EK, Moore AC, et al. (2009) Recombinant viral vaccines expressing merozoite surface protein-1 induce antibody- and T cell-mediated multistage protection against malaria. Cell Host Microbe 5: 95-105 |
|  | 390 | Jobe O, Donofrio G, Sun G, Liepinsh D, Schwenk R, et al. (2009) Immunization with radiation-attenuated Plasmodium berghei sporozoites induces liver cCD8alpha+DC that activate CD8+T cells against liver-stage malaria. PLoS One 4: e5075. |
|  | 391 | Jones TR, Obaldia Nr, Gramzinski RA, Hoffman SL (2000) Repeated infection of Aotus monkeys with Plasmodium falciparum induces protection against subsequent challenge with homologous and heterologous strains of parasite. Am J Trop Med Hyg 62: 675-680. |
|  | 392 | Kaba SA, Brando C, Guo Q, Mittelholzer C, Raman S, et al. (2009) A nonadjuvanted polypeptide nanoparticle vaccine confers long-lasting protection against rodent malaria. J Immunol 183: 7268-7277. |
|  | 393 | Kaur A, Kinhikar AG, Singh PP (2004) Bioimmunotherapy of rodent malaria: co-treatment  with recombinant mouse granulocyte-macrophage colony-stimulating factor and an enkephalin fragment peptide Tyr-Gly-Gly. Acta Trop 91: 27-41. |
|  | 394 | Khan ZM, Vanderberg JP (1992) Specific inflammatory cell infiltration of hepatic schizonts in BALB/c mice immunized with attenuated Plasmodium yoelii sporozoites. Int Immunol 4: 711-718. |
|  | 395 | Khullar N, Sehgal S (1990) Use of adjuvants in modulating the behaviour of Plasmodium berghei. Indian J Exp Biol 28: 1112-1117. |
|  | 396 | Khusmith S, Sedegah M, Hoffman SL (1994) Complete protection against Plasmodium yoelii by adoptive transfer of a CD8+ cytotoxic T-cell clone recognizing sporozoite surface protein 2. Infect Immun 62: 2979-2983. |
|  | 397 | Kumar S, Good MF, Dontfraid F, Vinetz JM, Miller LH (1989) Interdependence of CD4+ T cells and malarial spleen in immunity to Plasmodium vinckei vinckei. Relevance to vaccine development. J Immunol 143: 2017-2023. |
|  | 398 | Lanar DE, Tine JA, de Taisne C, Seguin MC, Cox WI, et al. (1996) Attenuated vaccinia virus-circumsporozoite protein recombinants confer protection against rodent malaria. Infect Immun 64: 1666-1671. |
|  | 399 | Li S, Rodrigues M, Rodriguez D, Rodriguez JR, Esteban M, et al. (1993) Priming with recombinant influenza virus followed by administration of recombinant vaccinia virus induces CD8+ T-cell-mediated protective immunity against malaria. Proc Natl Acad Sci U S A 90: 5214-5218. |
|  | 400 | Ling IT, Ogun SA, Holder AA (1994) Immunization against malaria with a recombinant protein. Parasite Immunol 16: 63-67. |
|  | 401 | Marussig M, Renia L, Motard A, Miltgen F, Petour P, et al. (1997) Linear and multiple antigen peptides containing defined T and B epitopes of the Plasmodium yoelii circumsporozoite protein: antibody-mediated protection and boosting by sporozoite infection. Int Immunol 9: 1817-1824. |
|  | 402 | Mauduit M, Gruner AC, Tewari R, Depinay N, Kayibanda M, et al. (2009) A role for immune responses against non-CS components in the cross-species protection induced by immunization with irradiated malaria sporozoites. PLoS One 4: e7717. |
|  | 403 | McColm AA, Bomford R, Dalton L (1982) A comparison of saponin with other adjuvants for the potentiation of protective immunity by a killed Plasmodium yoelii vaccine in the mouse. Parasite Immunol 4: 337-347. |
|  | 404 | McColm AA, Dalton L (1983) Heterologous immunity in rodent malaria: comparison of the degree of cross-immunity generated by vaccination with that produced by exposure to live infection. Ann Trop Med Parasitol 77: 355-377. |
|  | 405 | Mueller AK, Camargo N, Kaiser K, Andorfer C, Frevert U, et al. (2005) Plasmodium liver stage developmental arrest by depletion of a protein at the parasite– host interface. Proc Natl Acad Sci U S A 102: 3022-3027. |
|  | 406 | Orjih AU, Nussenzweig RS (1980) Immunization against rodent malaria with cryopreserved irradiated sporozoites of Plasmodium berghei. Am J Trop Med Hyg 29: 343-347. |
|  | 407 | Patterson PS, Bosshardt SC, Udhayukumar V, Xiao L, Kidd M, et al. (1999) Prolonged  expression of IFNgamma induced by protective blood-stage immunization against Plasmodium yoelii malaria. Vaccine 18: 173-180. |
|  | 408 | Perlaza B-L, Anais Zully Valencia, Constanza Zapata, Ange´lica Castellanos, Jean- Pierre Sauzet, et al. (2008) Protection against Plasmodium falciparum challenge induced in Aotus monkeys by liver-stage antigen-3- derived long synthetic peptides. Eur J Immunol 38: 2610–2615. |
|  | 409 | Roberts DW, Rank RG, Weidanz WP, Finerty JF (1977) Prevention of recrudescent malaria in nude mice by thymic grafting or by treatment with hyperimmune serum. Infect Immun 16: 821-826 |
|  | 410 | Playfair JH, De Souza JB (1979) Antibody responses in mice protected against malaria by vaccination. Parasite Immunol 1: 197-208. |
|  | 411 | Playfair JH, De Souza JB, Cottrell BJ (1977) Protection of mice against malaria by a killed vaccine: differences in effectiveness against P. yoelii and P. berghei. Immunology 33: 507-515. |
|  | 412 | Potocnjak P, Yoshida N, Nussenzweig RS, Nussenzweig V (1980) Monovalent fragments (Fab) of monoclonal antibodies to a sporozoite surface antigen (Pb44) protect mice against malarial infection. J Exp Med 151: 1504-1513. |
|  | 413 | Puri SK, Dutta GP, Levy HB, Maheshwari RK (1996) Poly ICLC inhibits Plasmodium cynomolgi B malaria infection in rhesus monkeys. J Interferon Cytokine Res 16: 49-52. |
|  | 414 | Pye D, O'Brien CM, Franchina P, Monger C, Anders RF (1994) Plasmodium falciparum infection of splenectomized and intact Guyanan Saimiri monkeys. J Parasitol 80: 558-562. |
|  | 415 | Reed RC, Louis-Wileman V, Wells RL, Verheul AF, Hunter RL, et al. (1996) Reinvestigation  of the circumsporozoite protein-based induction of sterile immunity against Plasmodium berghei infection. Vaccine 14: 828-836. |
|  | 416 | Renggli J, Hahne M, Matile H, Betschart B, Tschopp J, et al. (1997) Elimination of P. berghei liver stages is independent of Fas (CD95/Apo-I) or perforin-mediated cytotoxicity. Parasite Immunol 19: 145-148. |
|  | 417 | Renia L, Grillot D, Marussig M, Corradin G, Miltgen F, et al. (1993) Effector functions of circumsporozoite peptide-primed CD4+ T cell clones against Plasmodium yoelii liver stages. J Immunol 150: 1471-1478. |
|  | 418 | Reyes-Sandoval A, Berthoud T, Alder N, Siani L, Gilbert SC, et al. (2010) Prime-boost immunization with adenoviral and modified vaccinia virus Ankara vectors enhances the durability and polyfunctionality of protective malaria CD8+ T-cell responses. Infect Immun 78: 145-153. |
|  | 419 | Rodrigues EG, Claassen J, Lee S, Wilson JM, Nussenzweig RS, et al. (2000) Interferon-gamma-independent CD8+ T cell-mediated protective anti-malaria immunity elicited by recombinant adenovirus. Parasite Immunol 22: 157-160. |
|  | 420 | Rodrigues MM, Cordey AS, Arreaza G, Corradin G, Romero P, et al. (1991) CD8+ cytolytic T cell clones derived against the Plasmodium yoelii circumsporozoite protein protect against malaria. Int Immunol 3: 579-585. |
|  | 421 | Romero JF, Ciabattini A, Guillaume P, Frank G, Ruggiero P, et al. (2009) Intranasal administration of the synthetic polypeptide from the C-terminus of the circumsporozoite protein of Plasmodium berghei with the modified heat-labile toxin of Escherichia coli (LTK63) induces a complete protection against malaria challenge. Vaccine 27: 1266-1271. |
|  | 422 | Romero JF, Eberl G, MacDonald HR, Corradin G (2001) CD1d-restricted NK T cells are dispensable for specific antibody responses and protective immunity against liver stage malaria infection in mice. Parasite Immunol 23: 267-269. |
|  | 423 | Sadoff JC, Ballou WR, Baron LS, Majarian WR, Brey RN, et al. (1988) Oral Salmonella  typhimurium vaccine expressing circumsporozoite protein protects against malaria. Science 240: 336-338. |
|  | 424 | Schmidt LH, Rossan RN, Fradkin R, Sullivan R, Schulemann W, et al. (1985) Antimalarial activities and subacute toxicity of RC-12, a 4-amino-substituted pyrocatechol. Antimicrob Agents Chemother 28: 612-625. |
|  | 425 | Schneider J, Gilbert SC, Blanchard TJ, Hanke T, Robson KJ, et al. (1998) Enhanced  immunogenicity for CD8+ T cell induction and complete protective efficacy of malaria DNA vaccination by boosting with modified vaccinia virus Ankara. Nat Med 4: 397-402. |
|  | 426 | Schneider J, Gilbert SC, Blanchard TJ, Hanke T, Robson KJ, et al. (1998) Enhanced immunogenicity for CD8+ T cell induction and complete protective efficacy of malaria DNA vaccination by boosting with modified vaccinia virus Ankara. Nat Med 4: 397-402. |
|  | 427 | Sedegah M, Brice GT, Rogers WO, Doolan DL, Charoenvit Y, et al. (2002) Persistence of protective immunity to malaria induced by DNA priming and poxvirus boosting: characterization of effector and memory CD8(+)-T-cell populations. Infect Immun 70: 3493-3499. |
|  | 428 | Sedegah M, Finkelman F, Hoffman SL (1994) Interleukin 12 induction of interferon gamma-dependent protection against malaria. Proc Natl Acad Sci U S A 91: 10700-10702. |
|  | 429 | Siddiqui WA, Tam LQ, Kan SC, Kramer KJ, Case SE, et al. (1986) Induction of protective immunity to monoclonal-antibody-defined Plasmodium falciparum antigens requires strong adjuvant in Aotus monkeys. Infect Immun 52: 314-318. |
|  | 430 | Spitalny GL, Nussenzweig RS (1973) Plasmodium berghei: relationship between protective immunity and anti-sporozoite (CSP) antibody in mice. Exp Parasitol 33: 168-178. |
|  | 431 | Taylor-Robinson AW, Phillips RS (1994) Th1 and Th2 CD4+ T cell clones specific for Plasmodium chabaudi but not for an unrelated antigen protect against blood stage P. chabaudi infection. Eur J Immunol 24: 158-164. |
|  | 432 | Taylor-Robinson AW, Phillips RS, Severn A, Moncada S, Liew FY (1993) The role of TH1 and TH2 cells in a rodent malaria infection. Science 260: 1931-1934. |
|  | 433 | Tsuji M, Miyahira Y, Nussenzweig RS, Aguet M, Reichel M, et al. (1995) Development of antimalaria immunity in mice lacking IFN-gamma receptor. J Immunol 154: 5338-5344. |
|  | 434 | Trimnell A, Takagi A, Gupta M, Richie TL, Kappe SH, et al. (2009) Genetically attenuated parasite vaccines induce contact-dependent CD8+ T cell killing of Plasmodium yoelii liver stage-infected hepatocytes. J Immunol 183: 5870-5878. |
|  | 435 | van Dijk MR, Douradinha B, Franke-Fayard B, Heussler V, van Dooren MW, et al. (2005) Genetically attenuated, P36p-deficient malarial sporozoites induce protective immunity and apoptosis of infected liver cells. Proc Natl Acad Sci U S A 102: 12194-12199. |
|  | 436 | Vinetz JM, Kumar S, Good MF, Fowlkes BJ, Berzofsky JA, et al. (1990) Adoptive transfer of CD8+ T cells from immune animals does not transfer immunity to blood stage Plasmodium yoelii malaria. J Immunol 144: 1069-1074. |
|  | 437 | Waki S, Takagi T, Suzuki M (1989) Acquirement of protective immunity in mice through infection with an attenuated isolate and its failure in parent virulent Plasmodium berghei. Parasitol Res 75: 614-618. |
|  | 438 | Wang R, Charoenvit Y, Corradin G, De La Vega P, Franke ED, et al. (1996) Protection against malaria by Plasmodium yoelii sporozoite surface protein 2 linear peptide induction of CD4+ T-cell and IFN-dependent elimination of infected hepatocytes. J Immunol 157: 4061-4067. |
|  | 439 | Wang R, Charoenvit Y, Corradin G, Porrozzi R, Hunter RL, et al. (1995) Induction of protective polyclonal antibodies by immunization with a Plasmodium yoelii circumsporozoite protein multiple antigen peptide vaccine. J Immunol 154: 2784-2793. |
|  | 440 | Weiss WR, Berzofsky JA, Houghten RA, Sedegah M, Hollindale M, et al. (1992) A T cell clone directed at the circumsporozoite protein which protects mice against both Plasmodium yoelii and Plasmodium berghei. J Immunol 149: 2103-2109. |
|  | 441 | White KL, Jarboe DL, Krzych U (1994) Immunization with irradiated Plasmodium berghei sporozoites induces IL-2 and IFN gamma but not IL-4. Parasite Immunol 16: 479-491. |
|  | 442 | Wykes MN, Zhou YH, Liu XQ, Good MF (2005) Plasmodium yoelii can ablate vaccine-induced long-term protection in mice. J Immunol 175: 2510-2516. |
|  | 443 | Pacheco ND, McConnell E, Beaudoin RL (1979) Duration of immunity following a single vaccination with irradiated sporozoites of Plasmodium berghei. Bull World Health Organ 57 Suppl 1: 159-163. |
|  | 444 | Nussenzweig RS, Vanderberg JP, Most H, Orton C (1969) Specificity of protective immunity produced by x-irradiated Plasmodium berghei sporozoites. Nature 222: 488-489. |
|  | 445 | Nussenzweig R, Vanderberg J, Most H (1969) Protective immunity produced by the injection of x-irradiated sporozoites of Plasmodium berghei. IV. Dose response, specificity and humoral immunity. Mil Med 134: 1176-1182. |
|  | 446 | Orjih AU, Cochrane AH, Nussenzweig RS (1982) Comparative studies on the immunogenicity of infective and attenuated sporozoites of Plasmodium berghei. Trans R Soc Trop Med Hyg 76: 57-61. |
|  | 447 | Renia L, Rodrigues MM, Nussenzweig V (1994) Intrasplenic immunization with infected hepatocytes: a mouse model for studying protective immunity against malaria pre-erythrocytic stage. Immunology 82: 164-168. |
|  | 448 | Favila-Castillo L, Monroy-Ostria A, Kobayashi E, Hirunpetcharat C, Kamada N, et al. (1996) Protection of rats against malaria by a transplanted immune spleen. Parasite Immunol 18: 325-331. |
|  | 449 | Butler NS, Schmidt NW, Harty JT (2010) Differential Effector Pathways Regulate Memory CD8 T Cell Immunity against Plasmodium berghei versus P. yoelii Sporozoites. J Immunol 184: 2528-2538. |
|  | 450 | Yoshida S, Araki H, Yokomine T (2010) Baculovirus-based nasal drop vaccine confers complete protection against malaria by natural boosting of vaccine-induced antibodies in mice. Infect Immun 78: 595-602. |
|  | 451 | Falae A, Combe A, Amaladoss A, Carvalho T, Menard R, et al. (2010) Role of Plasmodium berghei cGMP-dependent protein kinase in late liver stage development. J Biol Chem 285: 3282-3288. |
| **b** | 47 | Belnoue E, Costa FT, Frankenberg T, Vigario AM, Voza T, et al. (2004) Protective T cell immunity against malaria liver stage after vaccination with live sporozoites under chloroquine treatment. J Immunol 172: 2487-2495. |
|  | 48 | Mueller AK, Labaied M, Kappe SH, Matuschewski K (2005) Genetically modified Plasmodium parasites as a protective experimental malaria vaccine. Nature 433: 164-167. |
|  | 124 | Vaughan JA, Scheller LF, Wirtz RA, Azad AF (1999) Infectivity of Plasmodium berghei sporozoites delivered by intravenous inoculation versus mosquito bite: implications for sporozoite vaccine trials. Infect Immun 67: 4285-4289. |
|  | 128 | Douradinha B, van Dijk MR, Ataide R, van Gemert GJ, Thompson J, et al. (2007) Genetically attenuated P36p-deficient Plasmodium berghei sporozoites confer long-lasting and partial cross-species protection. Int J Parasitol 37: 1511-1519. |
|  | 129 | Putrianti ED, Silvie O, Kordes M, Borrmann S, Matuschewski K (2009) Vaccine-like immunity against malaria by repeated causal-prophylactic treatment of liver-stage Plasmodium parasites. J Infect Dis 199: 899-903 |
|  | 138 | Ting LM, Gissot M, Coppi A, Sinnis P, Kim K (2008) Attenuated Plasmodium yoelii lacking purine nucleoside phosphorylase confer protective immunity. Nat Med 14: 954-958. |
|  | 171 | Labaied M, Harupa A, Dumpit RF, Coppens I, Mikolajczak SA, et al. (2007) Plasmodium yoelii sporozoites with simultaneous deletion of P52 and P36 are completely attenuated and confer sterile immunity against infection. Infect Immun 75: 3758-3768. |
|  | 387 | Aly AS, Mikolajczak SA, Rivera HS, Camargo N, Jacobs-Lorena V, et al. (2008) Targeted deletion of SAP1 abolishes the expression of infectivity factors necessary for successful malaria parasite liver infection. Mol Microbiol 69: 152-163. |
|  | 452 | Sina BJ, Wright C, Atkinson CT, Ballou R, Aikawa M, et al. (1995) Characterization of a sporozoite antigen common to *Plasmodium falciparum* and *Plasmodium berghei*. Mol Biochem Parasitol 69: 239-246. |
|  | 453 | Sina BJ, Wright C, Ballou R, Hollingdale M (1992) A protective monoclonal antibody with dual specificity for Plasmodium falciparum and Plasmodium berghei circumsporozoite proteins. Exp Parasitol 74: 431-440. |
| **c,x** | 124 | Vaughan JA, Scheller LF, Wirtz RA, Azad AF (1999) Infectivity of Plasmodium berghei sporozoites delivered by intravenous inoculation versus mosquito bite: implications for sporozoite vaccine trials. Infect Immun 67: 4285-4289. |
| **d** | 36 | Wizel B, Houghten R, Church P, Tine JA, Lanar DE, et al. (1995) HLA-A2–restricted cytotoxic T lymphocyte responses to multiple Plasmodium falciparum sporozoite surface protein 2 epitopes in sporozoite-immunized volunteers. J Immunol 155: 766-775. |
|  | 44 | Wizel B, Houghten RA, Parker KC, Coligan JE, Church P, et al. (1995) Irradiated sporozoite vaccine induces HLA-B8-restricted cytotoxic T lymphocyte responses against two overlapping epitopes of the Plasmodium falciparum sporozoite surface protein 2. J Exp Med 182: 1435-1445. |
|  | 118 | Clyde DF, Most H, McCarthy VC, Vanderberg JP (1973) Immunization of man against sporozite-induced falciparum malaria. Am J Med Sci 266: 169-177. |
|  | 119 | Clyde DF (1975) Immunization of man against falciparum and vivax malaria by use of attenuated sporozoites. Am J Trop Med Hyg 24: 397-401. |
|  | 120 | Clyde DF, McCarthy VC, Miller RM, Hornick RB (1973) Specificity of protection of man immunized against sporozoite-induced falciparum malaria. Am J Med Sci 266: 398-403. |
|  | 121 | Rieckmann KH, Carson PE, Beaudoin RL, Cassells JS, Sell KW (1974) Sporozoite induced immunity in man against an Ethiopian strain of Plasmodium falciparum. Trans R Soc Trop Med Hyg 68: 258-259. |
|  | 122 | Rieckmann KH, Beaudoin RL, Cassells JS, Sell KW (1979) Use of attenuated sporozoites in the immunization of human volunteers against falciparum malaria. Bull World Health Organ 57 Suppl 1: 261-265. |
|  | 123 | Rieckmann KH (1990) Human immunization with attenuated sporozoites. Bull World Health Organ 68 Suppl: 13-16. |
|  | 124 | Vaughan JA, Scheller LF, Wirtz RA, Azad AF (1999) Infectivity of Plasmodium berghei  sporozoites delivered by intravenous inoculation versus mosquito bite: implications for sporozoite vaccine trials. Infect Immun 67: 4285-4289. |
|  | 134 | Hoffman SL, Goh LM, Luke TC, Schneider I, Le TP, et al. (2002) Protection of humans against malaria by immunization with radiation-attenuated Plasmodium falciparum sporozoites. J Infect Dis 185: 1155-1164. |
|  | 135 | McCarthy VC, Clyde DF (1977) Plasmodium vivax: correlation of circumsporozoite precipitation (CSP) reaction with sporozoite-induced protective immunity in man. Exp Parasitol 41: 167-171. |
|  | 136 | Edelman R, Hoffman SL, Davis JR, Beier M, Sztein MB, et al. (1993) Long-term persistence of sterile immunity in a volunteer immunized with X-irradiated Plasmodium falciparum sporo-zoites. J Infect Dis 168: 1066-1070. |
|  | 172 | Palmer DR, Krzych U (2002) Cellular and molecular requirements for the recall producing memory CD4(+)CD45RO(+)CD27(-) T cells during protection induced attenuated Plasmodium falciparum sporozoites. Eur J Immunol 32: 652-661. |
|  | 454 | Clyde DF (1990) Immunity to falciparum and vivax malaria induced by irradiated sporozoites: a review of the University of Maryland studies, 1971-75. Bull World Health Organ 68 Suppl: 9-12. |
|  | 455 | Egan JE, Hoffman SL, Haynes JD, Sadoff JC, Schneider I, et al. (1993) Humoral immune responses in volunteers immunized with irradiated Plasmodium falciparum sporozoites. Am J Trop Med Hyg 49: 166-173. |
|  | 456 | Herrington DA, Nardin EH, Losonsky G, Bathurst IC, Barr PJ, et al. (1991) Safety and immunogenicity of a recombinant sporozoite malaria vaccine against Plasmodium vivax. Am J Trop Med Hyg 45: 695-701. |
| **e** | 111 | Chakravarty S, Cockburn IA, Kuk S, Overstreet MG, Sacci JB, et al. (2007) CD8+ T lymphocytes protective against malaria liver stages are primed in skin-draining lymph  nodes. Nat Med 13: 1035-1041. |
|  | 132 | Wong KA, Zhou A, Rodriguez A (2008) Protective immunity induced by daily bites from irradiated mosquitoes infected with Parasite Immunol 30: 482-486. |
|  | 457 | Schmidt NW, Butler NS, Harty JT (2009) CD8 T cell immunity to Plasmodium permits generation of protective antibodies after repeated sporozoite challenge. Vaccine 27: 6103-6106. |
| **f** | 127 | Sina BJ, do Rosario VE, Woollett G, Sakhuja K, Hollingdale MR (1993) Plasmodium falciparum sporozoite immunization protects against Plasmodium berghei sporozoite infection. Exp Parasitol 77: 129-135. |
| **g** | 163 | Roestenberg M, McCall M, Hopman J, Wiersma J, Luty AJ, et al. (2009) Protection against a malaria challenge by sporozoite inoculation. N Engl J Med 361: 468-477. |
| **h** | 48 | Mueller AK, Labaied M, Kappe SH, Matuschewski K (2005) Genetically modified Plasmodium parasites as a protective experimental malaria vaccine. Nature 433: 164-167. |
|  | 111 | Chakravarty S, Cockburn IA, Kuk S, Overstreet MG, Sacci JB, et al. (2007) CD8+ T lymphocytes protective against malaria liver stages are primed in skin-draining lymph nodes. Nat Med 13: 1035-1041. |
|  | 388 | Aly AS, Downie MJ, Mamoun CB, Kappe SH (2010) Subpatent infection with Nucleoside Transporter 1-deficient Plasmodium blood stage parasites confers sterile protection against lethal malaria in mice. Cell Microbiol. Epub ahead of print |
|  | 458  x2 | Butcher GA, Mitchell GH, Cohen S (1978) Antibody mediated mechanisms of immunity to malaria induced by vaccination with Plasmodium knowlesi merozoites. Immunology 34: 77-86. |
|  | 459 | Hommel M, David PH, Guillotte M, Pereira da Silva L (1982) Protection against Plasmodium  chabaudi malaria. I.--Vaccination of mice with merozoites and Freund's adjuvants. Ann Immunol (Paris) 133C: 57-67. |
