# Supplementary figures and images for: Why Functional Pre-Erythrocytic and Bloodstage Malaria Vaccines Fail: A Meta-Analysis of Fully Protective Immunizations and Novel Immunological Model
Source: PLoS One. 2010 May 19;5(5):e10685. doi: 10.1371/journal.pone.0010685 (PMC2873430; doi:10.1371/journal.pone.0010685)

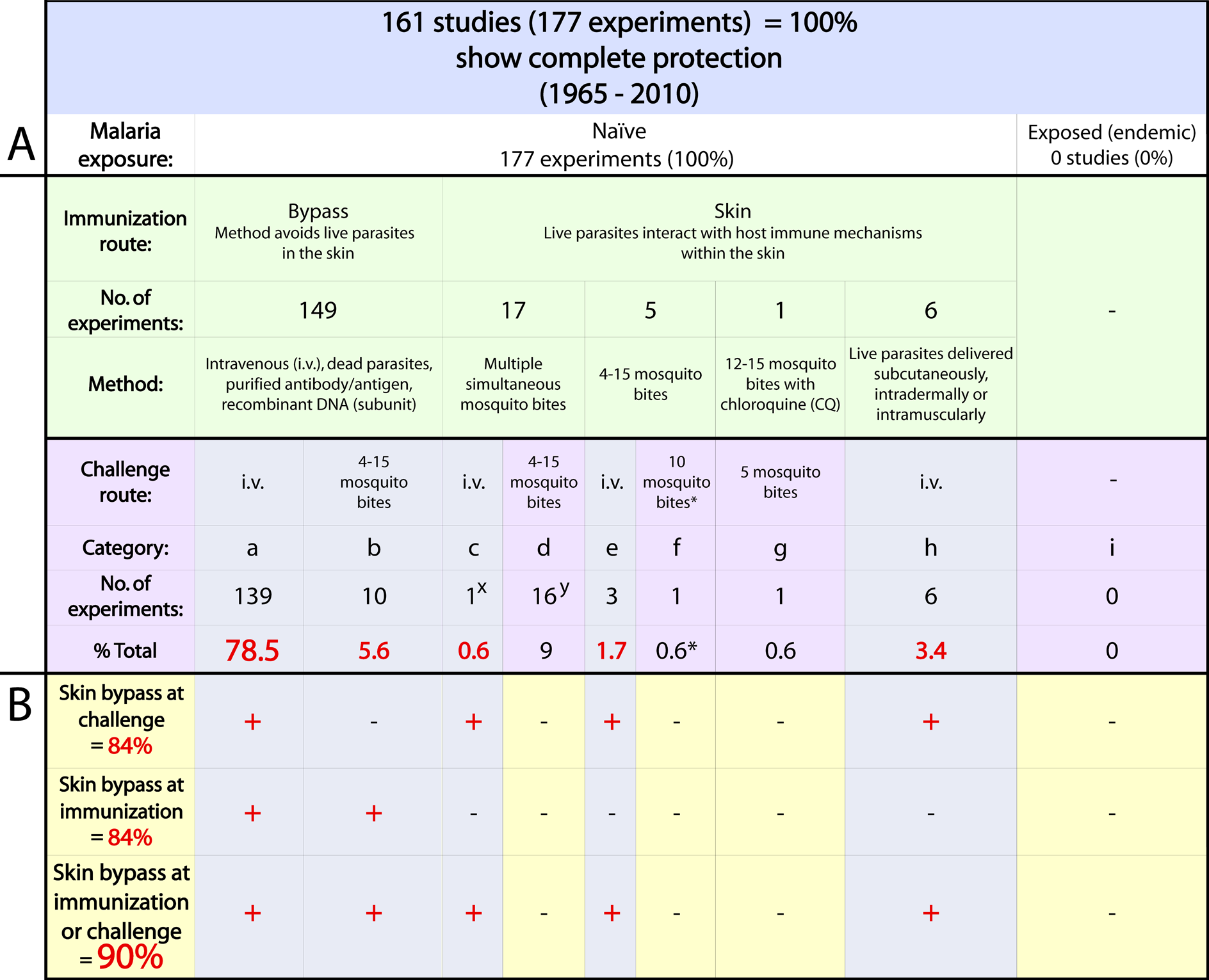

Supplement: Table S2 — Protective vaccination physically bypasses the skin at immunization or challenge (90%) or involves skin immunomodulation (10%). A. Exposure to parasites in the skin coincides closely with vaccine failure. Green background- immunization procedures. Lilac background- challenge procedures and percent of total experiments showing complete protection (% total) formed by a subset of studies (category) using a given experimental procedure (categories a-i; supporting experimental data for each category is in references listed below; also Supplementary Table S1). Parasite immunization administration routes: (a,b): live parasites given intravenously (i.v.), or the method does not involve live parasites, but uses dead parasites or purified antigen, antibody, or recombinant DNA (subunit) and therefore bypasses parasite interactions with host skin; (c,d,e,f,g,h): live parasites are administered via the skin; (c,d): by multiple simultaneous mosquito bites/session; (e,f): live parasites are naturally transmitted by 4–15 bites; (g): live parasites are administered by 12–15 bites/session with chloroquine; (h): live parasites delivered subcutaneously (s.c) or intradermally (i.d.) or intramuscularly (i.m.); (i): uncontrolled exposure to endemic mosquitos. Challenge route is either i.v. or by mosquito bite, as indicated. B. Protective immunization bypasses the skin at either immunization or challenge in 90% of cases: categories (a,b,c,e,h) shaded blue with red crosses. Protective immunization which transits skin during immunization (c,d,e,f,g,h) either: bypasses the skin physically at challenge (c,e,h) (red cross); or, involves skin immunomodulation during immunization (d,g, 10% of cases). Asterisk (*)- immunization via unmodified skin, limited to P. berghei (f). Skin bypass (red cross)- method physically avoids live parasite interactions in the host skin. Malaria exposure- skin exposure to infected mosquito bite before first immunization; naïve- no pre-exposure; exposed (endemic)- chr [file pone.0010685.s002.tif]
