# Supplementary material for: Why Functional Pre-Erythrocytic and Bloodstage Malaria Vaccines Fail: A Meta-Analysis of Fully Protective Immunizations and Novel Immunological Model
Source: PLoS One. 2010 May 19;5(5):e10685. doi: 10.1371/journal.pone.0010685 (PMC2873430; doi:10.1371/journal.pone.0010685)
Supplement: Text S1 — Antigen persistence in liverstages is not required for protection. Directly contradictory data [165], [166], [171], [267] is also easily reconciled. Like increasing irradiation [149], [165], the drug primaquine (PQ) eliminates liverstages [47], [166]. PQ however, also disrupts membrane and vesicular trafficking [159], [268], temporarily eliminating all antigen presentation. This prevents immunity if used during immunization [166], creating an apparent correlation between protection and parasite persistence [166]. Used after intravenous immunization [129], allowing early liverstage antigen presentation, however, multiple PQ-cure cycles provide sufficient cumulative antigen presentation to build immunity, without antigen persistence. (0.07 MB DOC) [file pone.0010685.s003.doc]

**Supplementary text**

**S1- Antigen persistence in liverstages is not required for protection**

Directly contradictory data [165,166,171,267] is also easily reconciled. Like increasing irradiation [149,165], the drug primaquine (PQ) eliminates liverstages [47,166]. PQ however, also disrupts membrane and vesicular trafficking [159,268], temporarily eliminating all antigen presentation. This prevents immunity if used during immunization [166], creating an apparent correlation between protection and parasite persistence [166]. Used after intravenous immunization [129], allowing early liverstage antigen presentation, however, multiple PQ-cure cycles provide sufficient cumulative antigen presentation to build immunity, without antigen persistence.
